# Modeling hippocampal spatial cells in rodents navigating in 3D environments

**DOI:** 10.1038/s41598-024-66755-x

**Published:** 2024-07-19

**Authors:** Azra Aziz, Bharat K. Patil, Kailash Lakshmikanth, Peesapati S. S. Sreeharsha, Ayan Mukhopadhyay, V. Srinivasa Chakravarthy

**Affiliations:** 1 Computational Neuroscience Lab, Indian Institute of Technology Madras, Chennai, 600036 India; 2 Department of Physics, Indian Institute of Technology Madras, Chennai, 600036 India; 3 Center for Complex Systems and Dynamics, Indian Institute of Technology Madras, Chennai, 600036 India; 4 Department of Biotechnology, Indian Institute of Technology Madras, Chennai, 600036 India; 5 Present Address: Instituto de Física, Pontificia Universidad Católica de Valparaíso, Valparaíso, Chile

**Keywords:** Autoencoders, 3D spatial cells, Head direction tuning, Lattice maze, Helical maze, Pegboard maze, Neuroscience, Computational neuroscience

## Abstract

Studies on the neural correlates of navigation in 3D environments are plagued by several issues that need to be solved. For example, experimental studies show markedly different place cell responses in rats and bats, both navigating in 3D environments. In this study, we focus on modelling the spatial cells in rodents in a 3D environment. We propose a deep autoencoder network to model the place and grid cells in a simulated agent navigating in a 3D environment. The input layer to the autoencoder network model is the HD layer, which encodes the agent’s HD in terms of azimuth (θ) and pitch angles (ϕ). The output of this layer is given as input to the Path Integration (PI) layer, which computes displacement in all the preferred directions. The bottleneck layer of the autoencoder model encodes the spatial cell-like responses. Both grid cell and place cell-like responses are observed. The proposed model is verified using two experimental studies with two 3D environments. This model paves the way for a holistic approach using deep neural networks to model spatial cells in 3D navigation.

## Introduction

Spatial navigation is a competency that is crucial for an organism's survival. A sizeable body of literature that seeks to study neural substrates for spatial navigation focuses on the hippocampus, thanks to the popularity of this system as the “GPS of the brain"^1–4^. Neuroscience research dedicated to spatial navigation and hippocampus has invested a far greater effort in 2D navigation than in 3D navigation, perhaps to the practical constraints involved in the study of the latter^5,6, 7^. The recordings of hippocampal neurons that potentially encode space are frequently taken from surface-dwelling animals like rats^8–10^ and only less often in flying creatures like bats^11–13^ to understand spatial maps in 3D.

Concepts that are relatively well established in the case of 2D navigation run into rough weather when extended to the 3D case. For example, the basis of neural encoding of head direction (HD) in 3D space still needs to be clarified, as experimental studies from different species yield conflicting reports. Shinder and Taube^14^ proposed that the HD cells of rats from the anterodorsal thalamus are mainly tuned to the horizontal directions, and tuning is intact as long as the pitch angle is less than 90 degrees. On the contrary, Laurens and Angelaki^15^ countered the claim and proposed that the HD cells are tuned in all three dimensions of angular space. Finkelstein et al. and Angelaki et al.^16,17^ proposed that the HD is tuned to azimuth and pitch/roll or the conjunctive combination of directions in bats. These discrepancies in results can be due to the distinctive navigational capabilities of rats and bats in their native environments^18^. Since bats fly, they map the environment volumetrically; hence, HD cells are tuned to combinations of azimuth and pitch or roll. Since rats generally dwell on the ground, their HD cell tunings are predominantly limited to azimuthal angles and less sensitively dependent on the pitch angle. Kim and Maguire^19^ demonstrated, using virtual reality (VR) experiments in humans, that the anterior thalamus and subiculum encode HD in the azimuthal plane while sensitivity for pitch directions is observed in the retro-splenial cortex.

The early discovery of place and grid cells in the hippocampus inspired an extensive search for a comprehensive cellular system in the hippocampus for encoding space. Many experimental studies in bats have reported 3D place cells^12,20–22^. On the contrary, reports of grid cells in bats from the entorhinal cortex are relatively rare^13,23^. Moreover, the grid cells in bats were recorded as the bat crawls on a 2D surface, and the gridness score was comparable with the grid cells of rats in a 2D environment. Therefore, there is a dearth of volumetric studies for grid cells in 3D, as mentioned above, which is the only experimental study to record from such cells in bats.

Experimental studies on rats in 3D environments show limited place cell and grid cell mapping on the z-axis (gravity axis)^8,10^. Grieves et al.^8^ showed that the place fields are elongated along the maze axes, but more fields in the vertical axis are significantly elongated compared to the other two axes in an aligned lattice maze (aligned to the ground). While in the case of a tilted lattice maze, the place fields were equally elongated in all three axes. The observation's rationale was that the animal was not moving freely in the gravity axis for the aligned lattice. In contrast, the motion was equiprobable for all axes in the tilted lattice. Similarly, Hayman and colleagues^10^ reported the elongation of place and grid fields on the gravity axis in pegboard and helical mazes.

In this study, we focus on developing a versatile deep autoencoder-based network to model grid and place cells in helical and pegboard mazes along with place cells in lattice mazes.

Although there are several computational models of hippocampal spatial cells concerning 2D environments^24–28^, very few models are available for 3D navigation. Mathis et al.^29^ described a mathematical model to show that the Face Centered Cubic (FCC) lattice is the most optimal representation for equivalent grid cells in a 3D environment. A computational study^30^ suggests the possibility of forming grid cells in FCC or Hexagonal Closed Packing (HCP) type of lattice configuration. Since the oscillatory interference models have intrinsic theta oscillations, and theta rhythms are not observed in freely flying bats, Horiuchi and Moss^31^ proposed a four-ring attractor network. The network uses the interference of spatial patterns from the ring attractors to construct grid patterns in 3D, assuming a reference vector separated by 109 degrees. Soman et al.^32^ proposed an anti-Hebbian network to model place cells in freely flying bats and studied the effect of the range of pitch angles on the isotropic nature of place cells and grid cells. The model's neurons also imitated border cells and theorized the presence of *plane* *cells*. Ginosar et al.^23^ proposed a model on recently discovered locally ordered grid cells in bats. Wang et al.^33^ proposed an oscillatory interference model on various 3D environments for rodents modelling grid cells, although the model is not trainable.

Deep learning is a widely accepted technique to solve a plethora of problems in diverse fields, including computer vision^34^, natural language processing^35^, reinforcement learning^36^, etc. It has proven its ascendency for object recognition tasks (Rashid et al. 2020)^37^. For a long time, the relevance of deep network models like Convolutional Neural Networks (CNNs) for neuroscience research has been thought to be limited to learning input–output behaviour. However, recent studies have shown that even the activation patterns of the layers of CNNs can be gainfully compared to the activation patterns of cortical areas in primary visual and auditory systems^38^. These modelling studies have inspired applying deep learning networks to model spatial cells in 2D and related navigational problems^39–41^. However, a paucity of studies applies deep learning techniques to model hippocampal responses in 3D.

We propose an autoencoder-based deep network to study spatial cells in rats navigating a 3D environment. Our model's path integration (PI) process is inspired by^32,28^, but an averaging process discards the time dependency of neural responses in the model^38^. The high-frequency components from the PI neurons in the model are removed using a low pass filter to make it purely a function of space. The grid and place cell responses emerge in the hidden layer of the model. Gong and Yu^42^ developed a 2D and 3D grid cell model considering 6 degrees of freedom (DOF) during navigation. They used a training-free recurrent neural network (RNN) that extends the idea of continuous attractor neural networks (CANN).

Our autoencoder-based deep neural network can generate grid cells and place cell-like firing responses. Instead of CANN, we start with oscillatory inputs inspired by oscillatory interference (OI) models. The desired activity of neurons arises automatically from network dynamics while training the model. Amongst all the available models of spatial cells in 3D environments, a few have attempted to design a comprehensive model to account for various experimental studies in 3D navigation. This paper primarily models two distinctive experimental studies^8,10^, each having two setups, an aligned and tilted lattice maze and a pegboard and helical maze.

The outline of the paper is as follows. The “Methods” section starts with data generation regarding trajectories used in different environments. Then the model architecture and the training procedure are described. The subsequent section describes the simulated results from two experimental studies^8,10^ and their statistical inferences. Lastly, we summarize the study in the discussion section.

## Methods

The proposed study aims to model the 3D spatial navigation experiments of^8,10^. In this study, we use Python programming language for modelling purposes and MATLAB for plotting and statistical analysis of results. First, we generate 2D trajectories, to classify our neurons as grid and place cells. The size of 2D arena for lattice maze is 5⨯5 units, 2⨯2 units for helical maze and 10⨯10 units for pegboard maze. Trajectories in a 2D environment are generated as described in^32,28, 38^. The classified neurons are further tested on the different 3D environments. These experimental studies in 3D use mazes with different geometries, which are described below.

### Trajectory generation for 3D lattice maze

**Figure 1:**
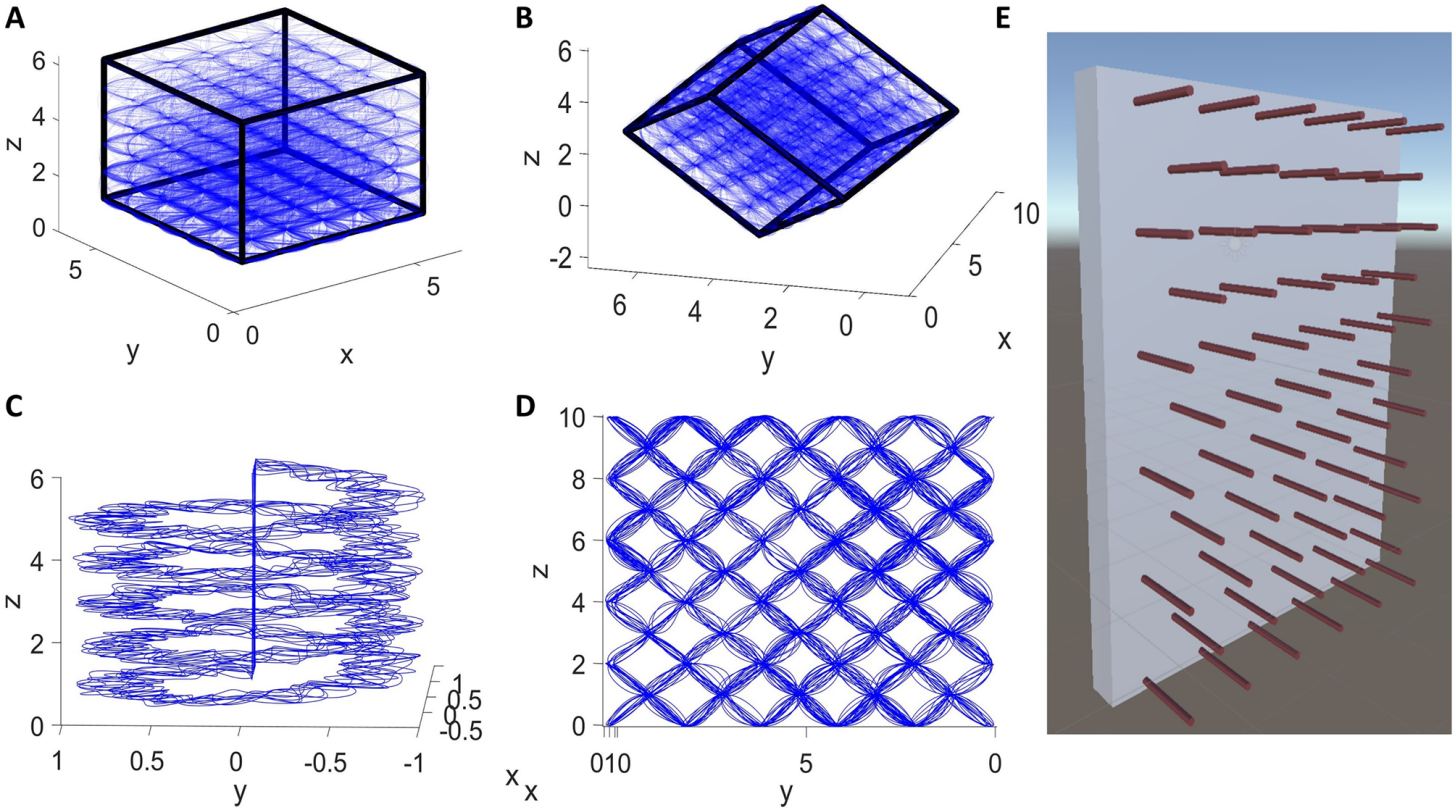
The virtual agent’s trajectory in (**A**) aligned lattice maze (**B**) tilted lattice maze. (**C**) Helical maze. (**D**) Pegboard maze. (**E**) Depicts the pegboard environment redrawn based on^10^.

To simulate the study of^8^ which uses a cubical lattice maze, we use a lattice maze of an outer boundary size of 5 × 5 × 5 units. Furthermore, navigation experiments were conducted under two conditions of the lattice: aligned lattice and tilted lattice (Fig. 1). Each axis has six bars, and the bars intersect to form nodes. The simulated agent's movement from one node to the other is based on probabilities similar to experimental conditions^8^, where the rat moved predominantly parallel to the X or Y axis (ground-axes) and moved less in the vertical dimension (see Supplementary material: Sect. 1) in case of aligned lattice maze (Fig. 1A). When the agent moves from one node to the other, it mostly moves within its 180 degrees horizontal field of view. The agent moves in the vertical direction (upwards or downwards) with a probability of 20% and on the horizontal axis with a probability of 80%. In a tilted lattice maze, the agent moves with equal probabilities on all three axes (Fig. 1B, see Supplementary material: Sect. 1). The resulting sequence of nodes was smoothened using a cubic spline to form a smooth trajectory. Speed is assumed constant throughout, and T number of equidistant points are generated along the trajectory.

### Trajectory generation for helical maze

The helical trajectory is generated with an assumption of a virtual agent similar to the lattice maze. This trajectory consists of five coils starting from the ground and extending to a height of five units, increasing uniformly throughout the helix. The inner radius of the path for each coil is 0.5 units, and the outer radius is 1 unit. In this path, the animal can move freely in any random trajectory within the above bounds (Fig. 1C). These dimensions match the experimental conditions^10^. Moreover, the upward and downward trajectories are generated separately. For upward motion, the agent directly returns to the ground after reaching the top without any downward movement along the helix, and vice versa for the downward motion.

### Trajectory generation for pegboard maze

A trajectory on a pegboard of dimensions 1 $\times$ 10 $\times$ 10 units is created. Here the peg length is 1 unit, whereas the pegboard is 10 $\times$ 10 units (Fig. 1D and E). The horizontal and vertical distance between two pegs is 2 units. Therefore, the distance from a peg to the neighboring diagonal peg is √2. The agent moves in the YZ plane with equal probability from any given peg to neighboring diagonal pegs with the restriction that it will not return to the peg it just came from. The X-axis coordinates (along the peg) are chosen randomly using a random number generator between 0 and 1. The agent hops 2000 times from peg to peg. Then these trajectory points are interpolated to produce 100,000 points and smoothed using spline functions.

### Model description

#### Head direction (HD) layer

HD matrix represents the preferred directions of neurons in the azimuth ($\theta$) and pitch ($\phi$) plane. Here we consider n_1_ number of equally spaced azimuth angles spanning 360 degrees and n_2_ number of equally spaced pitch angles spanning 180 degrees. Using these angles, (n_1_ $\times$ n_2_) unit vectors were used as the preferred directions of the HD neurons. The matrix of preferred directions (Eq. 1) is calculated as follows.

1 $$\begin{aligned}{U}_{i,x} & =sin({\phi }_{i})cos({\theta }_{i}) \\ {U}_{i,y} & =sin({\phi }_{i})sin({\theta }_{i}) \\{U}_{i,z} & =cos({\phi }_{i}) \\ {U}_{i} & =[{U}_{i,x }{U}_{i,y} {U}_{i,z}] \end{aligned}$$

where, ${U}_{i,x},{U}_{i,y},{U}_{i,z}$ are the components of HD in x, y and z axes for *ith* direction. The response of the *i*th HD cell, HD_*i*_ (Eq. 2), is given as

2 $$H{D}_{i}=v \cdot {U}_{i}$$

where *v* is the velocity of the agent in 3D space and *U*_*i*_ is the preferred direction.

#### PI layer

In the PI layer, the neural responses are functions of space and time^32,28^. As the spatial cell responses are typically depicted in terms of space alone, we remove the time dependency from the PI neurons by an averaging process as discussed in^38^ (Supplementary material: Sect. 8). The neurons in the PI and the HD layers have a one-to-one connection. The response of the ith neuron of the PI layer is given as (Eq. 3),

3 $$\begin{aligned}P{I}_{i} & =cos(\beta {\int }_{0}^{t}H{D}_{i}dt) \\ P{I}_{i}& =cos(\beta {\int }_{0}^{t}v \cdot {U}_{i}dt) \\ P{I}_{i} & =cos(\beta z \cdot {U}_{i})\end{aligned}$$

where *z* is the displacement of the agent from the initial position and β is the modulation factor. The choice of $\beta$ depends on size of environment. The formulation of this PI can be extended to a complex form as ${PI}_{i} \approx {e}^{j(\beta Z \cdot {u}_{i})}$.

For the implementation, we use concatenation of sine and cosine terms as input to the autoencoder model i.e., concatenate ($cos\left(\beta z \cdot {U}_{i}\right) and sin(\beta z \cdot {U}_{i}))$^38^.

#### Autoencoder model

**Figure 2:**
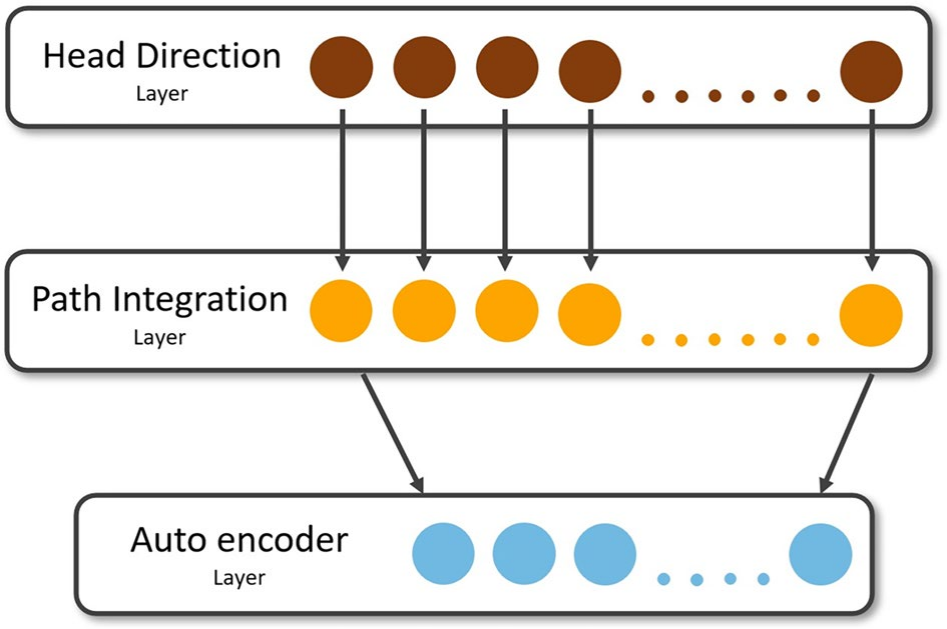
A schematic of the autoencoder model. Circles represent neurons.

The PCA model for grid cells^32,28, 38^, shows that the PI’s covariance matrix is circulant. It is well known that the eigenvectors of the circulant matrix are sinusoids, hence, giving rise to periodic firing in PI. The autoencoder works similarly to PCA with the additional capability of performing non-linear transformations. Therefore, we choose autoencoders for modeling the experimental data. The autoencoder is an unsupervised neural network model that reduces dimensionality^43^. The output from the PI layer is passed on to the autoencoder network through feedforward connections (Fig. 2). The hyperparameters used for training the autoencoder network are shown in Table 1 for all experiments.

**Table 1:** Parameters and hyperparameters used in the autoencoder network.

Parameters and hyperparameters	Value
Θ (azimuth angle)	0-2π
Φ (pitch angle)	0-π
n_1_ (no. of preferred azimuth angles)	20
n_2_ (no. of preferred pitch angles)	3
n (no. of hidden neurons in layer 1 and layer 2 of the autoencoder model)	50
Activation function	Linear
Data points (lattice and helix mazes)	50,000
Data points (pegboard maze)	100,000
Loss function	Mean square error (MSE)
Training: validation	4:1
Training epochs	10

### Place cell and grid cell classification

#### Place cell

To classify a neuron as place cell, we analyze the firing rate map of the neuron for two properties i.e., spatial information and sparsity^44^.

$$spatial \; info= \sum_{i=1}^{N}{{P}_{i}}_{\frac{{R}_{i}}{R}{log}_{2}\frac{{R}_{i}}{R}}$$

where, ${P}_{i}$ is the occupancy probability of the ith bin, ${R}_{i}$ is the mean firing rate for bin i, $R$ is the overall mean firing rate for the cell and N is total number of bins.

$$sparsity= \sum \frac{({P}_{i}*{{R}_{i}}^{2})}{{R}^{2}}$$

where, ${P}_{i}$ is the occupancy probability of the ith bin, ${R}_{i}$ is the mean firing rate of cell in i^th^ bin and R is the overall mean firing rate.

A random shuffling test is done on the neurons whose spatial information is greater than 0.3 bits/spikes and sparsity is less than 0.1. The neurons whose spatial information is above 99^th^ percentile and sparsity less than 1 percentile are classified as place cell.

#### Grid cell

To quantify the gridness score of hexagonal and square grid cells, autocorrelation is calculated using the equation^32,28^:

$$r\left(\tau x , \tau y\right)= \frac{M{\sum }_{x,y}\lambda \left(x,y\right)\lambda \left(x-{\tau }_{x},y-{\tau }_{y}\right)- {\sum }_{x,y}\lambda \left(x,y\right){\sum }_{x,y}\lambda \left(x-{\tau }_{x},y-{\tau }_{y}\right)}{\sqrt{[M {\sum }_{x,y}\lambda {\left(x,y\right)}^{2}-{[{\sum }_{x,y}\uplambda \left(x,y\right)]}^{2}]-[M{\sum }_{x,y}\lambda {\left(x-{\tau }_{x},y-{\tau }_{y}\right)}^{2}]-[{\sum }_{x,y}\lambda {(x-{\tau }_{x},y-{\tau }_{y})]}^{2}]}}$$

where, r is an autocorrelation map, $\lambda$ (x, y) is the firing rate at location (x, y) in firing rate map, M is the total number of pixels in the firing rate maps and τ_x_ and τ_y_ are the x and y coordinate spatial lags.

Using autocorrelation, Hexagonal Grid Score (HGS) and Square Grid Score (SGS) are calculated using the following equations:

$$HGS=min\left[cor\left(r,{r}^{{60}^{^\circ }}\right),cor\left(r,{r}^{{120}^{^\circ }}\right)\right] -max[cor\left(r,{r}^{{30}^{^\circ }}\right),cor\left(r,{r}^{{90}^{^\circ }}\right),cor\left(r,{r}^{{150}^{^\circ }}\right)]$$

$$SGS=cor\left(r,{r}^{{90}^{^\circ }}\right)-max[cor\left(r,{r}^{{45}^{^\circ }}\right),cor\left(r,{r}^{{135}^{^\circ }}\right)]$$

where, r^θ^ is the autocorrelation rotated by θ^°^. The neurons whose HGS and SGS are greater than 0.3 are classified as hexagonal grid cell and square grid cell. Throughout this study, we only consider the hexagonal grid cell and square grid cells are excluded.

## Results

### Simulation studies

The common underlying theme of the simulation studies described below is to train the model in the 2D environment. The response of the encoder layer from the autoencoder is analyzed for grid and place cells as described in the “Methods” section. These spatial cells are further tested on the 3D mazes and the results are reported. This study mainly focuses on two experimental paradigms^8,10^ therefore, we test the space encoding properties of place cells on lattice mazes (aligned and tilted) and place and grid cells on the helical and pegboard mazes.

### 3D lattice maze

**Figure 3:**
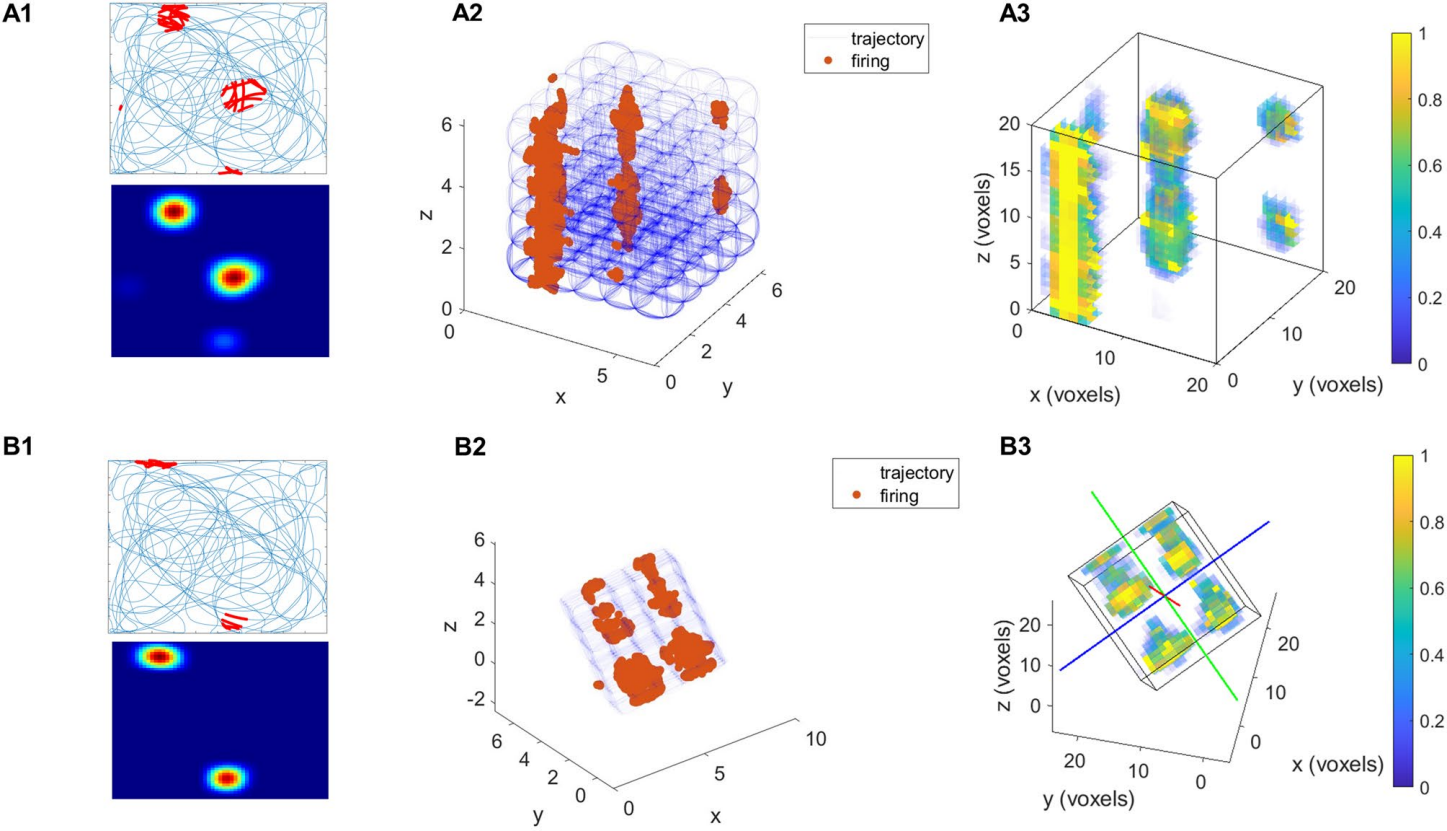
Firing field and firing rate map (**A1**) of a place cell in the flat arena. Firing field (**A2**) and firing rate map (**A3**) of corresponding place cell in the aligned lattice maze. (**B**) Similar to (**A**) for tilted lattice maze.

The simulations are done for two lattice mazes: aligned lattice and tilted lattice maze. The results are discussed simultaneously for both cases. For this study, we first train the autoencoder model with 2D trajectory, analyze the hidden layer response for place cells and then test these place cells on lattice mazes (Fig. 2). The output of these neurons is saved for analysis to understand the place cell responses in a volumetric environment. The raw output of the neurons is thresholded appropriately. Trajectory points where the activity of a neuron crosses the threshold are indicated by red dots (Fig. 3A1 and B1, Supplementary Fig. S1.1).

In both the mazes the aligned and tilted lattice, out of the 50 hidden neurons, 26% of neurons show responses similar to place cells in arena (“Methods”). The place and grid fields are oriented along the maze border. Out of all fields, 70% of grid fields and 73% of place fields in aligned and 80% of grid fields and 69% of place fields in tilted lattice mazes are oriented along the maze borders. These place cells are further tested in lattice mazes and analyzed using firing rate maps (Fig. 3A2 and B2, Supplementary Fig. S1.2).

To generate firing rate maps, the 5 $\times$ 5 $\times$ 5 unit^3^ environment is divided into 20 $\times$ 20 $\times$ 20 voxels. The firing activity is calculated using the thresholded data as specified in the experimental study^8^ (Eq. 4). The firing rate at the jth voxel position ${x}_{j}$ is defined as,

4 $$f\left({x}_{j}\right)= \frac{{\sum }_{i=1}^{n}g({X}_{ij}- {x}_{j})}{\sum_{t=1}^{T}g(y\left(t\right)- {x}_{j})}$$

where, ${X}_{ij}$ is the *ith* firing point location in the firing field in *jth* voxel, n is the total number of firing locations inside a voxel, $y\left(t\right)$ is the position of the rat at a discrete-time step *t*, T is the total number of trajectory points, ${x}_{j}$ is the position of the voxel center whose firing rate is being calculated. The truncated Gaussian function *g* gives non-zero values for neighboring 26 voxels.

Connected voxels are isolated by a MATLAB function “regionprops3” and are interpreted as place fields if the region is larger than 50 voxels (Fig. 3A2 and B2). Place cells are observed to have multiple place fields (Fig. 3A2 and B2) identical to the experimental study^8^. The properties of place fields for both aligned and titled lattice mazes are then screened for further analysis.

### Place fields were uniformly distributed in both mazes

**Figure 4:**
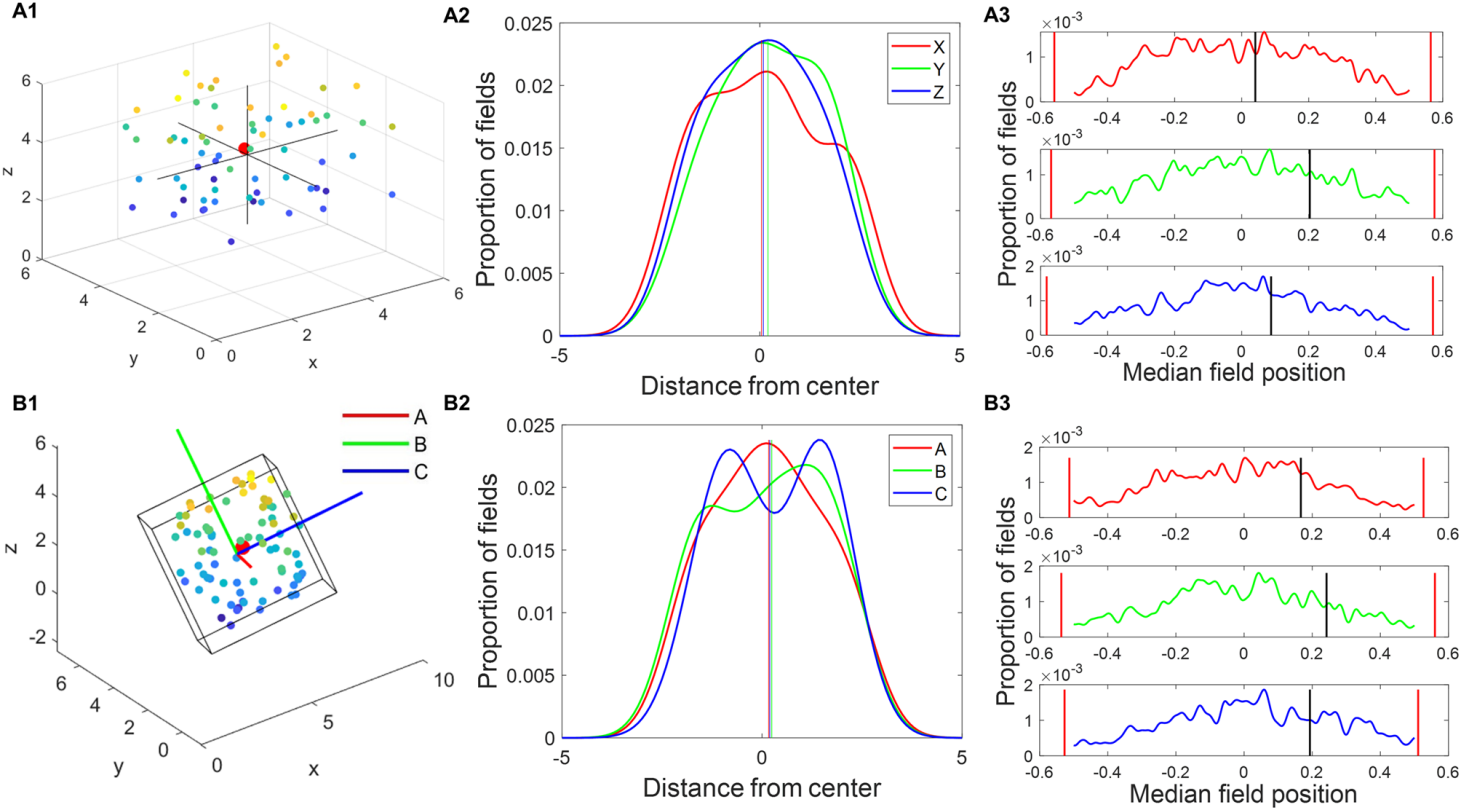
(**A**) represents the aligned lattice. (**A1**) Centroids of the place fields are represented by colored dots (hotness of plotted dots are directly proportional to the height in the maze. The red dot is the median of centroids of all place fields). (**A2**) Distribution of place fields along each axis inside the volume. Lines represent the median for each axis. (**A3**) Distribution created by generating N random points (equal to place field) and calculating their median for 1000 times. Red lines depict the 2.5th and 97.5th percentiles. Black lines represent the actual median of place fields. (**B**) Represents the same analysis for the tilted lattice case.

We reproduced the distribution results observed in the experimental analysis to check whether the firing fields were uniformly distributed. We observed that the median field centroids, generated by the autoencoder, lie close to the maze centers along each axis (Fig. 4A1 and B1). The median of centroids along each axis lies within the 95% confidence intervals of a shuffle distribution generated by taking N uniform random points (where N is the number of place fields screened) within the lattice frame and calculating their centroids (Fig. 4 and Supplementary material: section "3D lattice maze").

### Place fields were elongated

**Figure 5:**
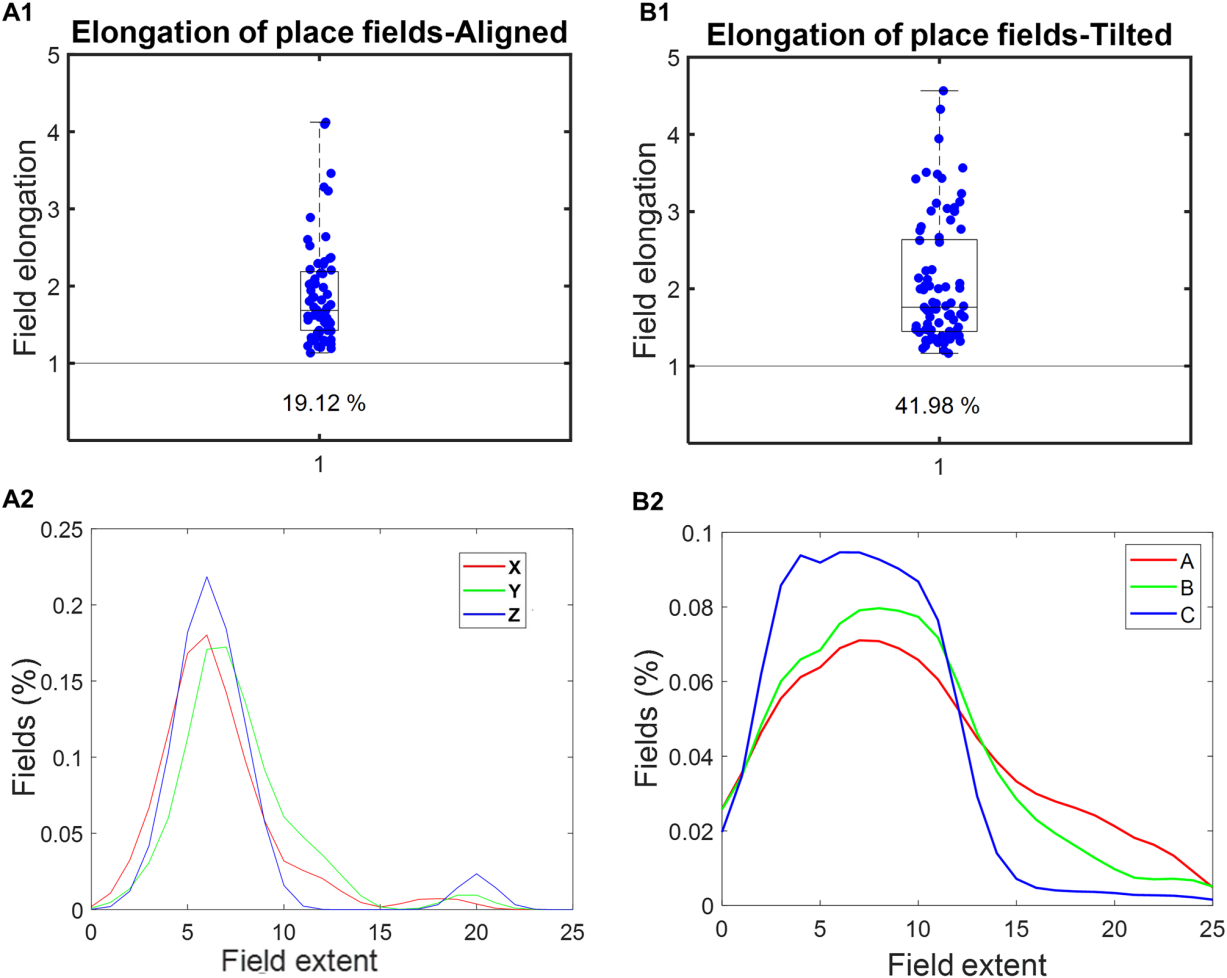
(**A**) For the aligned lattice. (1) The elongation indices of all place fields are represented by blue dots. Index value of 1 (gray line in box plot) indicates a spherical field. The text below the box plot represents the percentage of place fields that are equally or more spherical by chance. (2) Distribution for the proportion of cells for field length along the respective axis. Z-axis in the aligned lattice has a significantly different bimodal occurrence. All the other axes have similar distributions. (**B**) Represents the same analysis for the tilted lattice.

Most firing fields were elongated in both the lattice mazes. Most elongation indices (see Supplementary material: Sect. 4) deviated from 1 (elongation of 1 indicates a spherical place field). A small percentage (Fig. 5A1 and B1 text) of place fields were more spherical than would be expected by chance in both cases (Fig. 5A1 and B1).

The distribution of the proportion of fields showing the length of fields shared the same unimodal appearance across all axes in tilted and aligned except for the Z-axis in the case of the aligned lattice. Z-axis had bimodal occurrence with a second maximum for more extended fields. This extension showed a significant elongation along the Z-axis compared to the others (Fig. 5A2 and B2).

### Place fields were oriented parallel to the maze axes

**Figure 6:**
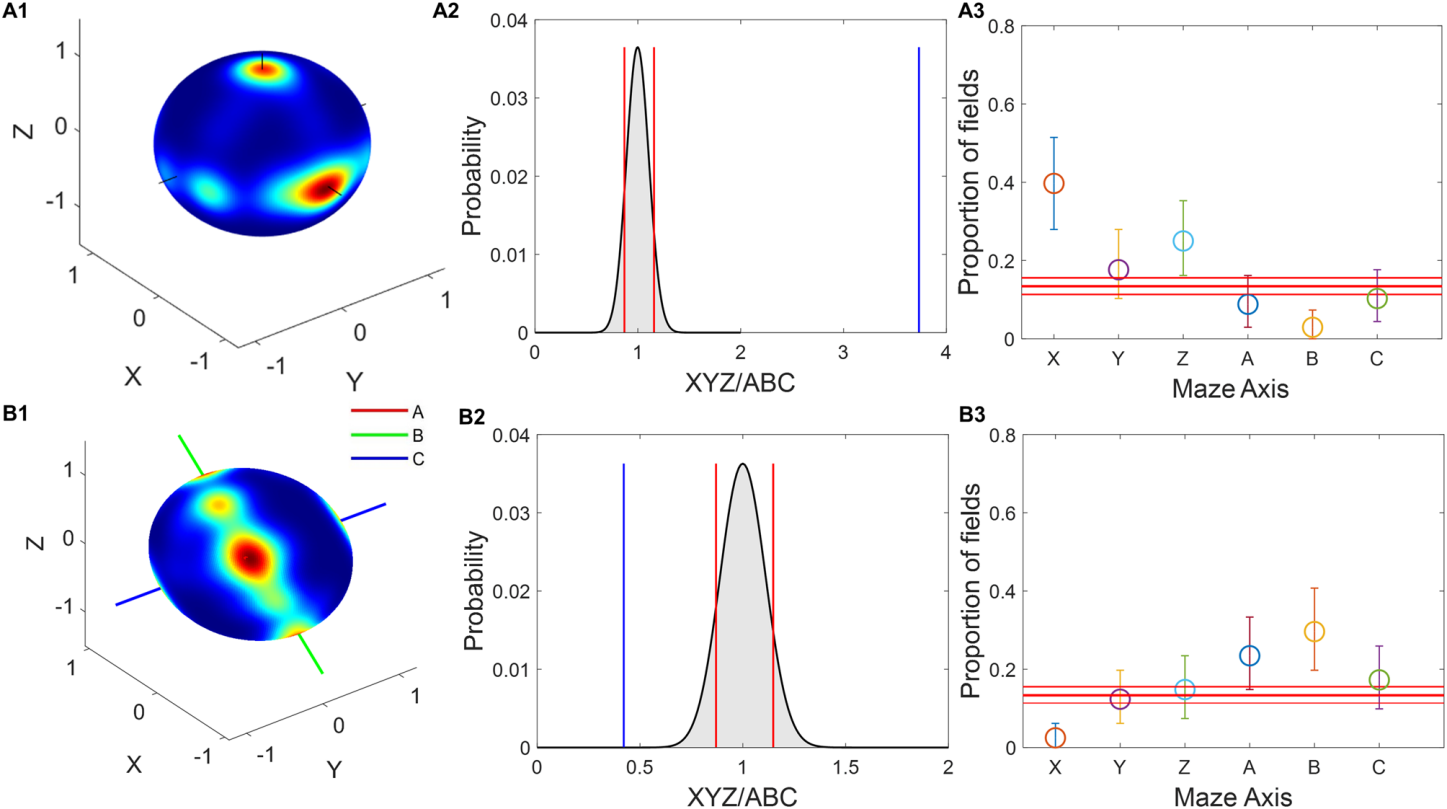
The place fields were oriented parallel to the maze axes. Hot colors represent the direction of the first principal axis of the place fields. (**A1**) Place field orientation plot for aligned lattice maze. (**B1**) Place field orientation plot for the tilted lattice maze. (**A2**) distribution of the ratio of two axes system using shuffle test. Red lines represent 1st and 99th percentiles, blue line represents the actual value for aligned lattice. (**A3**) Axis-wise orientation of the firing fields with 2.5th and 97.5th percentiles as error bars calculated using uniform sampling. The three red bars represent the 2.5th, 50th and 97.5th percentile of the firing fields expected by chance along any axis. (**B2**,**B3**) represent the same analysis for the tilted lattice.

As in the last section, it is shown that the place fields are elongated in nature. However, to understand the orientation of the elongated place fields, the orientation of the principal axis of the field is calculated and projected onto a unit sphere. Most fields are oriented along the maze cardinal axes in the aligned lattice maze (Fig. 6A1 and A3). In a tilted lattice maze, most fields are oriented along tilted axes, i.e., A, B, and C axes (Fig. 6B1). Figure 6A3 and B3 shows the orientation of firing fields along specific axes. In the aligned case, the firing field orientations for all three axes are more than expected by chance (97.5th percentile) (Fig. 6A3) similar to the original experiment^8^, where all three axes (X, Y and Z) have firing field orientations expected more than by chance. For the tilted lattice, the firing field orientations along A, B and C axes are more than expected by chance (97.5th percentile) (Fig. 6B3) and is comparable to the original experiment^8^. In aligned cases, YZ and AC axes are considered not different as they fall into the range of each other's error bars (Supplementary methods: Sect. 5—uniform sampling). For tilted lattice, The X, Y, and Z axes compared to A, B, and C axes are significantly different regarding field orientations (Supplementary methods: Sect. 5). To compare the field orientation between the cartesian and rotated systems, we calculated the ratio of field orientation (Fig. 6A2 and B2) along both systems. The observed results were found to be more than expected by chance (99th percentile) in the case of aligned lattice and less than expected by chance (1st percentile) for titled lattice (Fig. 6A2 and B2) (Supplementary material: Sect. 5). This shows that the firing fields are more oriented towards the cartesian system in the aligned lattice and the rotated system in the tilted lattice.

### Place fields were elongated parallel to the maze axes

The results above show that both mazes' firing fields are elongated along the maze axes. However, it is essential to know if there is a preferential elongation along any particular axis in both lattice mazes. To achieve this, a morphological erosion process was performed on the 3D binary volumetric image of firing fields using structuring element vectors of varying lengths along each cartesian axis (Supplementary Methods Sect. 4, Supplementary methods^8^). For each erosion operation, the linear sum of the remaining voxels to measure the map's connectivity along that dimension is first calculated. It is then expressed as the proportion of all remaining voxels for that element length. It is observed that place fields are elongated more along the vertical (Z) axis in the aligned lattice maze.

**Figure 7:**
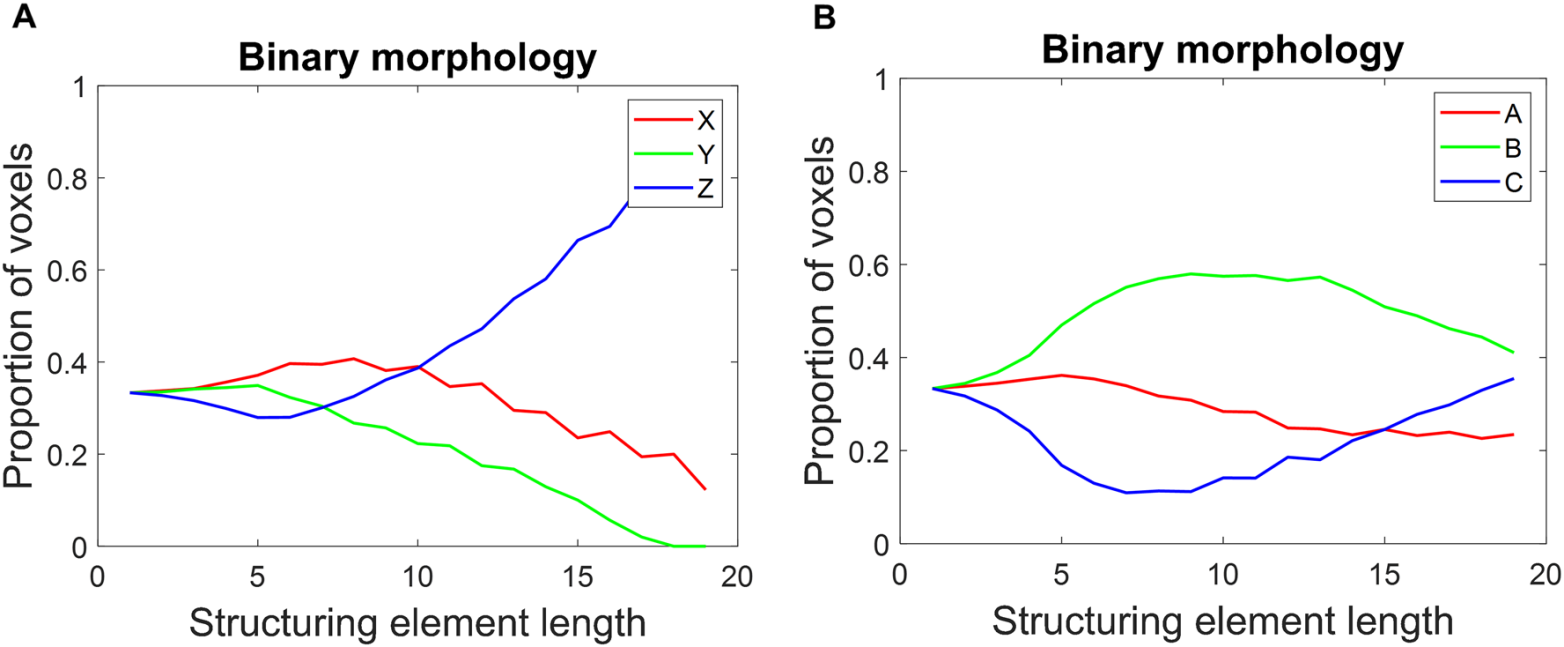
Binary morphological analysis of place cells' firing rate maps. (**A**) Initially, for smaller structuring element lengths, the connectivity is the same and then begins to diverge after erosion using structuring elements with lengths greater than 7. Hence, the voxels are highly connected along the Z-axis compared to the other cardinal axes, thereby suggesting a lower vertical spatial coding by place cells for the aligned lattice case. (**B**) Axes in tilted lattice show equivalent proportion of field elongated along all.

In contrast, no significant preference is seen in the tilted lattice maze, i.e., a nearly equal fraction of firing fields is elongated along all three axes (Fig. 7A and B). Hence, the spatial information conveyed by the place cells in an aligned lattice maze is less along the Z-axis in an aligned lattice maze. Using the binary morphology technique (Supplementary material: Sect. 6), we showed that vertical spatial coding was less accurate than along the horizontal axes of the maze in the aligned lattice.

### Helical maze and pegboard

Hayman et al.^10^ carried out two experimental studies to understand the response of grid cells and place cells in 3D environments in rodents. This study used two experimental setups: a pegboard maze setup and a helical maze setup. We use the autoencoder architecture described in “Methods” section (Fig. 2) to model the experimental results of^10^ with 20 azimuth and 1 pitch angle i.e., 90° (horizontal to the plane). We have used two sampled t-test for comparative studies, except mentioned otherwise.

### Helical maze

To simulate the results, we created helical trajectories with a five-coil helix for upward and downward movements separately.

### Training of the model

Like the lattice maze, we first train the model with the trajectory on a flat arena. The encoder layer contains fifty neurons. The grid and place cells are analyzed from the encoder layer, and these neurons are further tested on the Helical maze for upward and downward runs separately (Fig. 2). A neuron is considered to be "firing" if it crosses a specified threshold, and a red dot marks the location of its firing on a blue trajectory.

#### Results

In the Helical maze, a run is considered a traversal of the Helix from top to bottom or vice-versa. As the results are similar in both cases, we show a combined analysis of both traversals. Firstly, we train the autoencoder model in a flat arena. The 50 neurons in the encoder layer are analyzed for the grid and place cells (“Methods” section). Of 50 neurons, 18% are classified as grid cells, whereas 38% are classified as place cells. The same model is tested on the Helical maze, and the response of the grid and place cells (from the flat arena) in the helical maze are observed. We analyse the firing pattern of these neurons in both runs and flat arena. Among all grid and place fields, 85% grid fields and 73% place fields are oriented along the maze axes. The neural response for all the place and grid cell responses are shown in (Supplementary material: Sect. 7 Figs. S2 and S3).

**Figure 8:**
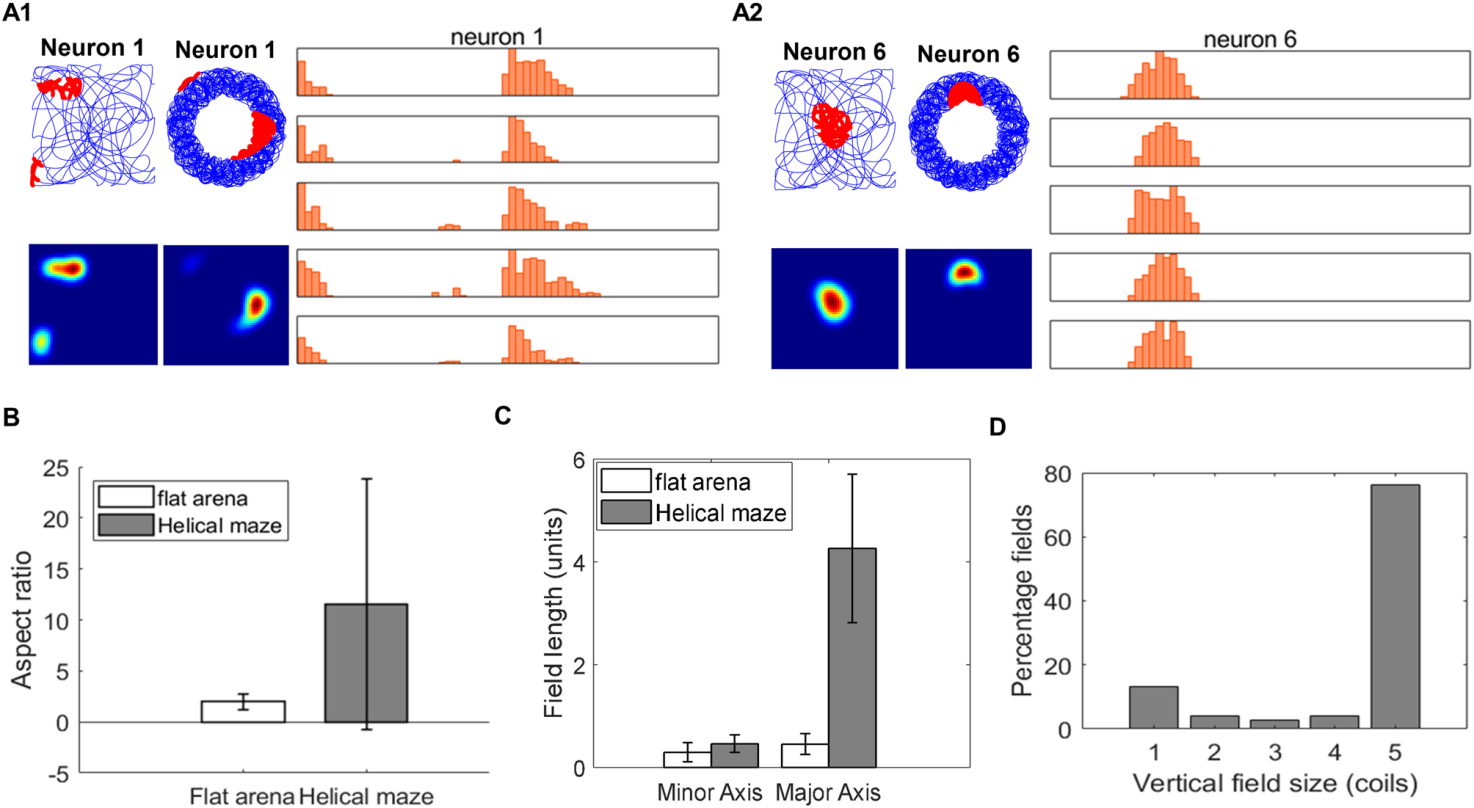
(**A1**) and (**A2**) depict the activities of two sample place cells. (**A1** and **A2**) The first column shows the place cell firing fields and firing rate maps on the flat arena. The middle column shows the top view of firing fields and firing rate map in the helical maze, and the right column denotes the linear firing frequency on each of the five coils as a function of position along the track (divided into 60 bins). (**B**) Aspect ratio of major and minor axes on the flat arena and helical maze. (**C**) Average field length along major and minor axes for flat arena and helical maze. (**D**) Percentage fields extending along the number of coils in the helical maze. The error bars in the figure shows the standard deviation for the respective plot.

A top view of the maze is plotted, showing localized firing fields of place cells. To analyze the firing field, each coil is unwound and divided into 60 bins to represent positions (Fig. 8A1 and A2). Multiple firing fields on a coil are observed, although the firing fields on multiple coils are at relatively similar (x, y) locations. The length of the firing field on the helical maze is calculated by considering the adjacent fields along the coils at similar (x, y) locations. The mean length of the minor axis of fields in the Helical maze (0.48 ± 0.18 units) is more significant than the mean length of the minor axis of fields in the 2d maze (0.32 ± 0.19 units; t110 = − 4.48, P < 0.0001). The mean length of the major axis of the fields (4.47 ± 1.19 units) is significantly greater than the mean length of the minor axis of the Helical maze (0.48 ± 0.18 units; t150 = − 28.81, P < 0.0001) (Fig. 8C). The mean aspect ratio of the fields in the Helical maze (11.28 ± 10.48 units) is significantly greater than the mean aspect ratio in the 2d maze (1.99 ± 0.13 units; t110 = 5.29, P < 0.0001), which is contrary to the observations in the experimental study^10^ (Fig. 8B). The reason for this discrepancy is that the size of the field on the (x, y) plane in the model depends on β. If the field is smaller in size in the (x, y) plane, the aspect ratio tends to be greater in the case of a Helical maze. The fields extending for five coils constitute about an 80%, whereas fields extending for less than five coils constitute 20% (Fig. 8D).

**Figure 9:**
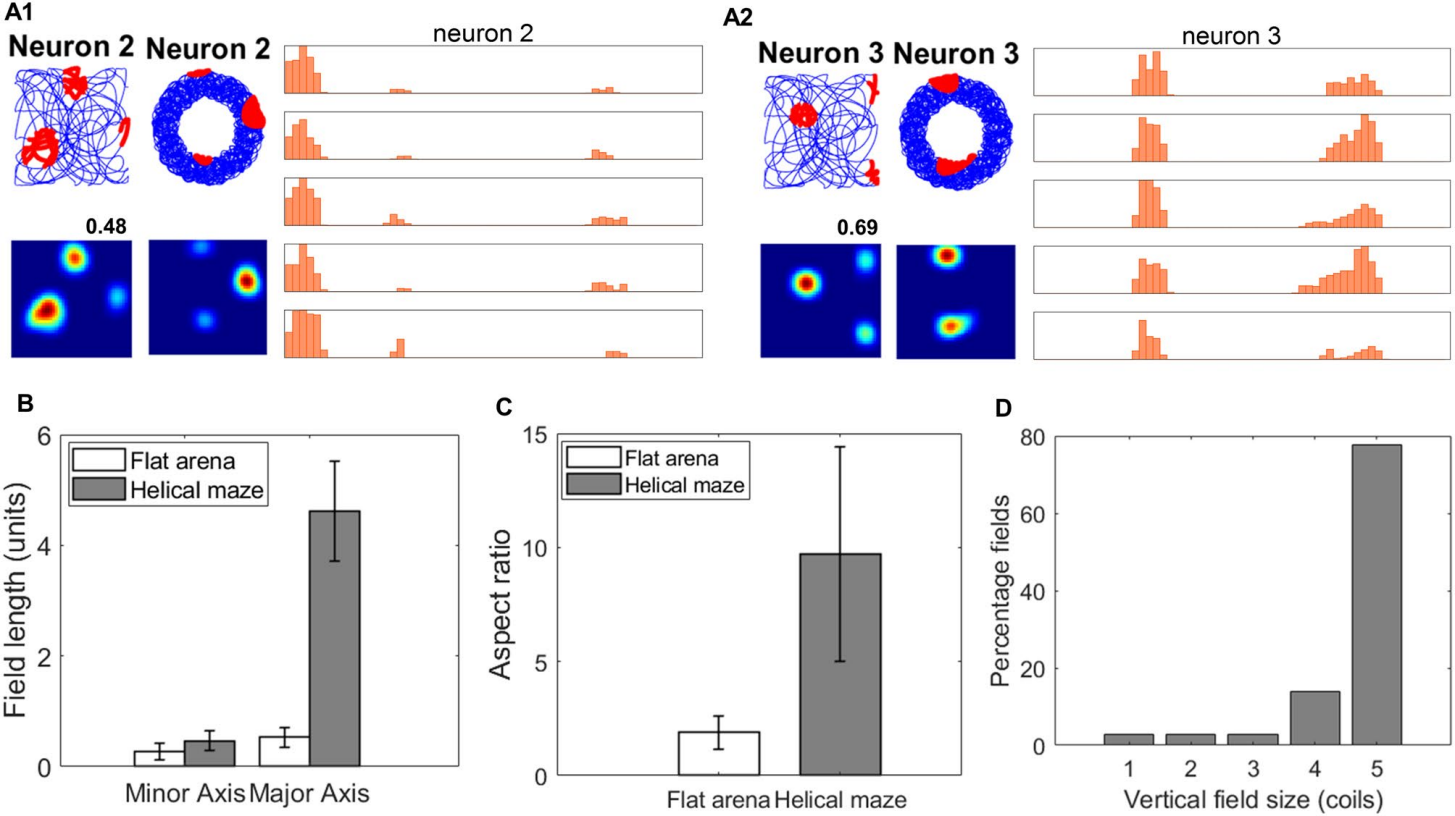
(**A1**) and (**A2**) depict the activities of two sample grid cells. (**A1** and **A2**) The first column shows the flat arena's grid cell firing fields and firing rate maps (the number on top of firing rate maps is the hexagonal grid score)*.* The middle column shows the top view of firing fields and firing rate map in the helical maze, and the right column denotes the linear firing frequency on each of the 5 coils as a function of position along the track (divided into 60 bins). (**B**) Average field length along major and minor axes for flat arena and helical maze. (**C**) Aspect ratio of major and minor axes on the flat arena and helical maze. (**D**) Percentage fields extending along the number of coils in the helical maze. The error bars in the figure shows the standard deviation for the respective plot.

We did a similar analysis for grid cells in the Helical maze. A top view of both the mazes is plotted to show grid cell response, and the helical maze coils are unwound (Fig. 9A1 and A2). The mean of the minor axis of fields in the Helical maze (0.53 ± 0.18 units) is more significant than the mean of the minor axis of fields in the 2d maze (0.2 ± 0.15 units; t63 = − 6.03, P < 0.0001). The mean length of the major axis of the fields (4.61 ± 0.90 units) is significantly greater than the mean length of the minor axis of the Helical maze (0.53 ± 0.18 units; t70 = − 26.61, P < 0.0001) (Fig. 9B). Similar to the place fields, the mean aspect ratio of the fields in the Helical maze (9.71 ± 4.71 units) is significantly greater than the mean aspect ratio of the fields in the 2d maze (1.88 ± 0.73 units; t63 = 8.86, P < 0.0001) (Fig. 9C). The fields extending for five coils constitute 77.8%, whereas fields extending for less than five coils constitute 22.8% (Fig. 9D).

### Pegboard maze

We created a trajectory on a vertical maze that has pegs protruding out of a vertical board (Fig. 1E). The agent moves around the vertical board with pegs as stepping stones.

### Training the model

We trained the autoencoder model in a flat arena and analyzed the encoder layer for grid and place cells. The neurons that pass the criteria of hexagonal grid cells and place cells (“Methods” section) are tested on the 3D pegboard environment.

#### Results

**Figure 10:**
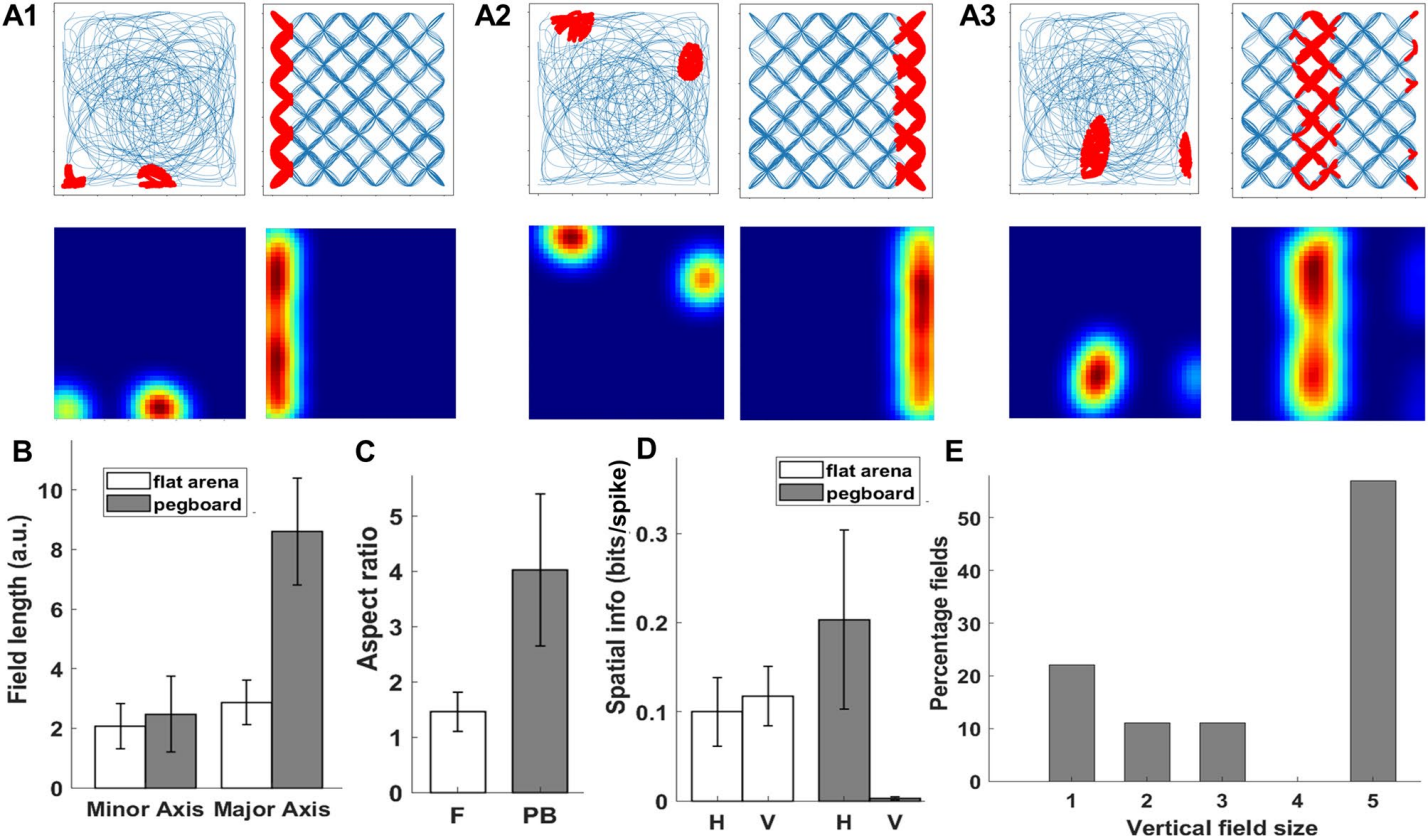
(**A1**,**A2**,**A3**) are three example place cells in the flat arena (left) and 3D pegboard environment (right). Top row shows the firing fields of place cells and bottom row shows the firing rate maps. (**B**) Average field length for major and minor axis of place field in flat arena and pegboard. (**C**) Aspect ratio of length of major and minor axis in flat arena and pegboard. (**D**) Spatial information along horizontal and vertical axes in flat arena and pegboard. (**E**) Percentage of place fields with their respective vertical field size. The error bars in the figure shows the standard deviation for the respective plot.

The encoder layer of the model contains 50 neurons, of which 20% (10 neurons) exhibited grid cells and 26% (13 neurons) exhibited place cell-like responses (Supplementary Sect. 8: Figs. S4 and S5). Place cells have an elongated elliptical firing field on the pegboard compared to slightly oval fields on the flat arena (Fig. 10A1, A2, and A3) and are oriented along the maze border. Among all grid and place fields, 85% of place fields and 82% of grid fields are oriented along the maze border in flat arena. The minor axis had no significant difference between the pegboard and the flat arena (F: 2.07 ± 0.76; PB: 2.47  ± 1.27; t_38_ = 1.26, p = 0.22). The major axis was significantly longer in the pegboard than in the flat arena (F: 2.87 ± 0.75; PB: 8.60  ± 1.79; t_38_ = 14.37, p < 0.0001) (Fig. 10B). The elongation of firing fields is deduced by calculating the aspect ratios (Major Axis/Minor Axis) for all the fields in both environments and comparing them to completely spherical fields (aspect ratio = 1, one sampled t-test). Firing fields in the flat arena were slightly oval (F: 1.46 ± 0.35; t_52_ = − 6.69, p < 0.0001), typical for place cells but were highly elongated in the pegboard (PB: 4.02 ± 1.37; t_24_ = − 7.92, p < 0.0001) (Fig. 10C). To check whether the fields were elongated vertically, we also compared spatial information on the x and y axes for flat and the horizontal and vertical axes for the pegboard. The firing rate map pixels were collapsed into a linear array by averaging them over all columns for vertical spatial information content and all rows for horizontal spatial information content. The horizontal and vertical information content was not different in the flat arena (Horizontal spatial information: 0.10  ± 0.04; Vertical Spatial information: 0.12  ± 0.03_;_ t_24_ = − 1.26, p = 0.22), but in pegboard, the information content was significantly lower in vertical axis compared to horizontal axis (Horizontal spatial information: 0.2 ± 0.10; Vertical Spatial information: 0.003 ± 0.002_;_ t_18_ = 6.32, p < 0.0001) (Fig. 10D). We also observed the expanse of fields on the vertical axis by dividing the pegboard into five different layers and manually counting the number of layers each field extended. Most place cells had fields extending over all five vertical layers (63%) (Fig. 10E) (Supplementary Sect. 8: Fig. S6).

**Figure 11:**
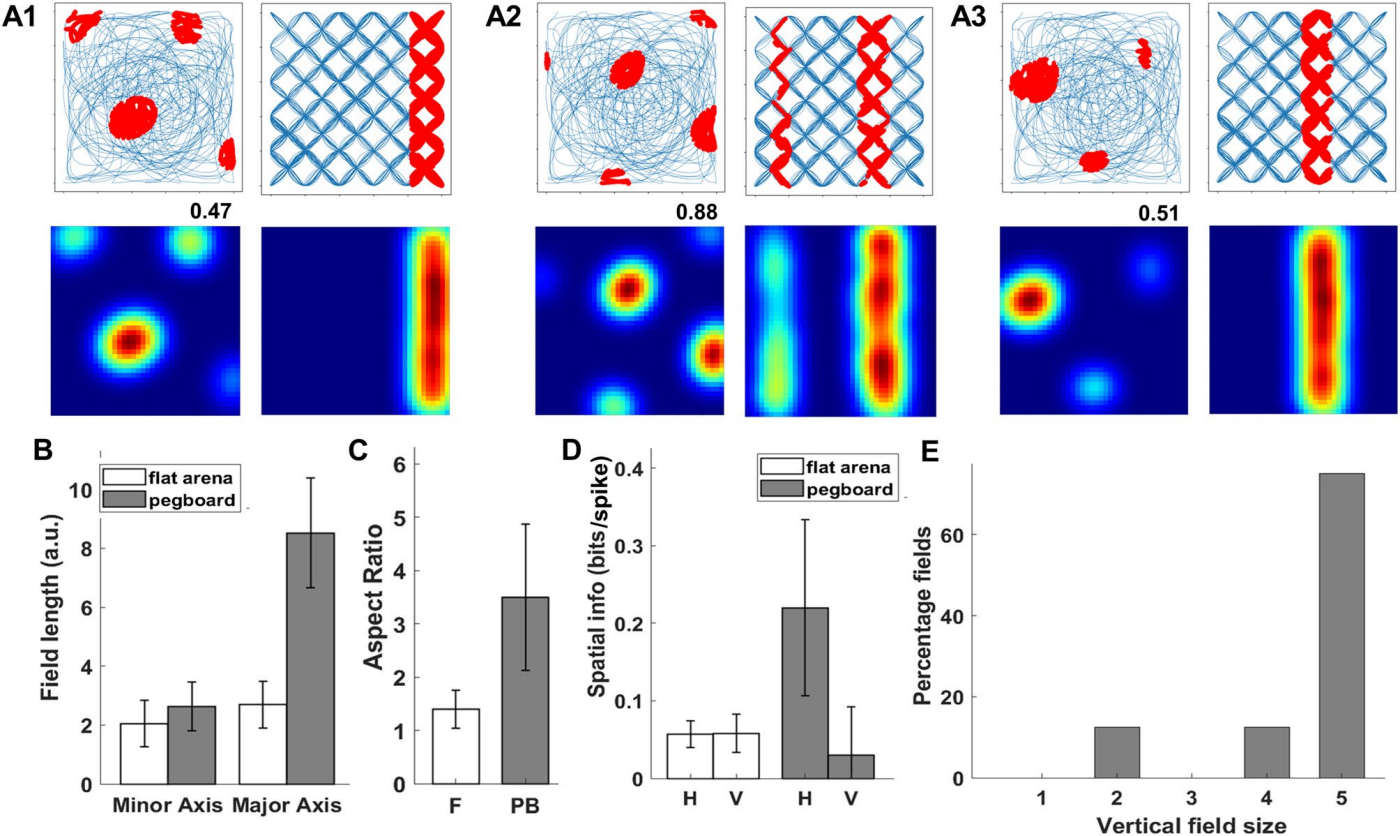
(**A1**,**A2**,**A3**) are three example grid cells in the flat arena (left) and 3D pegboard environment (right). Top row shows the firing fields of place cells and bottom row shows the firing rate maps. (**B**) Average field length for major and minor axis of grid field in flat arena and pegboard. (**C**) Aspect ratio of length of major and minor axis in flat arena and pegboard. (**D**) Spatial information along horizontal and vertical axes in flat arena and pegboard. (**E**) Percentage of grid fields with their respective vertical field size. The error bars in the figure shows the standard deviation for the respective plot.

We performed a similar analysis for neurons categorized as grid cells on a flat arena and tested them on a pegboard (Fig. 11A1–A3). The length of the minor axis did not differ significantly between the flat arena and pegboard (F: 2.05 ± 0.79; PB: 2.63 ± 0.83_;_ t_49_ = − 2.21, p = 0.03), but the major axis was significantly elongated in pegboard (F: 2.69 ± 0.79; PB: 8.51 ± 1.86_;_ t_49_ = − 15.69, p < 0.0001) (Fig. 11B). Similar to place cells, individual grid fields were slightly oval (F: 1.39 ± 0.36; t_38_ = − 6.92, p < 0.0001) on the flat arena but were highly elongated on the pegboard (PB: 3.50 ± 1.37; t_11_ = − 6.30, p < 0.0001), which is deduced by calculating the aspect ratio for all fields in both environments and comparing them to spherical fields (aspect ratio = 1, one sampled t-test) (Fig. 11C). We also calculated the horizontal and vertical spatial information for all the grid cells in both environments using a similar method for place cells. Grid cells too, did not have a significant difference in horizontal and vertical spatial information (Horizontal spatial information: 0.06 ± 0.02; Vertical Spatial information: 0.06 ± 0.03; t_20_ = − 0.13, p = 0.90), but on pegboard, the vertical information was significantly lower than horizontal information (Horizontal spatial information: 0.22 ± 0.11; Vertical Spatial information: 0.03 ± 0.06; t12 = 3.88, p < 0.001) (Fig. 11D). Most of the fields spanned through the entire length of the pegboard (75%), which was counted by dividing the pegboard into five layers and manually counting the expanse of individual fields (Fig. 11E) (Supplementary Sect. 8: Fig. S6).

## Discussions

Computational models of the hippocampal spatial cells in 3D navigation are scarce. The few existing models are mainly abstract theoretical models of spatial cells and do not try to reproduce experimental results^29–31^. One study^32,28^ that uses anti-Hebbian learning to model spatial cells like place, plane, and grid cells in case of bats navigating in 3D space. However, there is a dearth of comprehensive computational models that can model grid cells and place cells in diverse 3D environments. Hence, following the work of^32,28^ this study proposes an autoencoder-based deep learning approach to model place and grid cells in specific environmental paradigms. This approach has been successfully employed to model place and grid cells in a 2D environment^38^.

We simulate the experimental study^10^ that shows rodent place cell and grid cell firing patterns when foraging on Pegboard and Helix. When the autoencoder model is trained on a 2D track and further tested in a 3D environment for pegboard, we observe the emergence of grid cells and place cells in autoencoder layer similar to the original experiment^10^. The same was observed when we trained the model on a helical maze. The emerging spatial cells show stark spatial selectivity on horizontal planes and the elongation observed along the vertical plane. We also observe mixed responses of place and grid cells in the autoencoder layer. The deviation in the aspect ratio of the major and minor axis in pegboard and helix results from the experimental results is due to choosing the value of β. A larger value of β gives smaller-sized fields and vice versa. Therefore, to get a larger minor axis in a helical maze, we can choose a smaller β.

The other study we stimulated was from^8^, which involved a rat’s movement along an aligned and tilted 3D lattice maze. Like pegboard and helical mazes, we first train the model on a 2D environment of size comparable to that of lattice mazes. Further, we simulate a trajectory that allows a rat to forage along a 3D lattice cube which can then be rotated to incorporate the settings from the original experimental study and use the rotated trajectory to test the autoencoder network. The current autoencoder network model can explain most of the experimental observations (emergence of place cells, distribution of place cells, elongation, orientation along axes, and preferential elongation along specific axis). The current paper only discusses place cells from the 3D lattice experimental study^8^.

It is shown in previous computational models of bat navigation that place cells in bats tend to have more isotropic fields. This model advances this previous work in two major ways: (1) it has been shown by^8^ that the higher proportion of fields were elongated along the gravity axis and thus have lower spatial information. This observation is speculated due to the trajectory distribution of rodents (lower accessibility to the gravity axis) which emerges from their restricted physiology in 3D compared to bats. The proposed model incorporates this by assuming the Z axis as the vertical/gravity axis and thereby limiting accessibility comparative to the horizontal axes (X and Y). The modelled cells exhibit this anisotropy, elongation and orientation in 2D/3D lattice maze. (2)^32,28^ uses Lateral Anti–Hebbian Network (LAHN) to model place cells in 3D for bat and rodent navigation. This LAHN layer is updated to an autoencoder layer by^38^ which shows emergence of place and grid cells in 2D. This advancement paves a way for multisensory integration using realistic visual cues, real-time navigation using reinforcement learning signals and comprehensive modelling of most spatial cells such as vector-based navigation and memory. The current model sheds light on the capacity of simplistic models with a novel, simple path integration mechanism that can model a variety of cells^39^ and can also incorporate novel experimental findings such as the ones modelled in this manuscript.

We have successfully presented the versatile nature of the deep learning approach to model spatial cells in hippocampal formation. The proposed modelling approach is quite comprehensive as it uses autoencoder layer to model place and grid cells in different studies and environments using a single model.

### Supplementary Information

Supplementary Information.

## Data Availability

The datasets and code to generate results and data for this study can be found at the ModelDB database. (Model URL: https://modeldb.science/2016220, Access code: cnslab).

## Abbreviations

PI: Path integration
FCC: Face centered cubic
HCP: Hexagonal closed packing
CNN: Convolution neural networks
DOF: Degree of freedom
RNN: Recurrent neural network
CANN: Continuous attractor neural network
OI: Oscillatory interference
HD: Head direction
HGS: Hexagonal grid score
SGS: Square grid score
