# Supplementary material for: Modeling hippocampal spatial cells in rodents navigating in 3D environments
Source: Sci Rep. 2024 Jul 19;14:16714. doi: 10.1038/s41598-024-66755-x (PMC11271631; doi:10.1038/s41598-024-66755-x)
Supplement: Supplementary file 1 — Supplementary Information. [file 41598_2024_66755_MOESM1_ESM.docx]

Supplementary Material

1. Trajectory: Lattice maze (aligned and tilted)

Table 1: Probability of virtual agent choosing the direction of next node in aligned lattice maze

| Aligned Lattice Probabilities | |
| --- | --- |
| Preferred Movement | Probability |
| 1. Vertical movement (along the Z axis)    1. Upward movement    2. Downward movement | 20%  8%  12% |
| 1. Horizontal movement (along X and Y axes) | 80% |
| Tilted Lattice Probabilities | |
| Preferred Movement | Probability |
| 1. Movement along A, B and C axes | 33.33% |

1. Place cells and firing fields


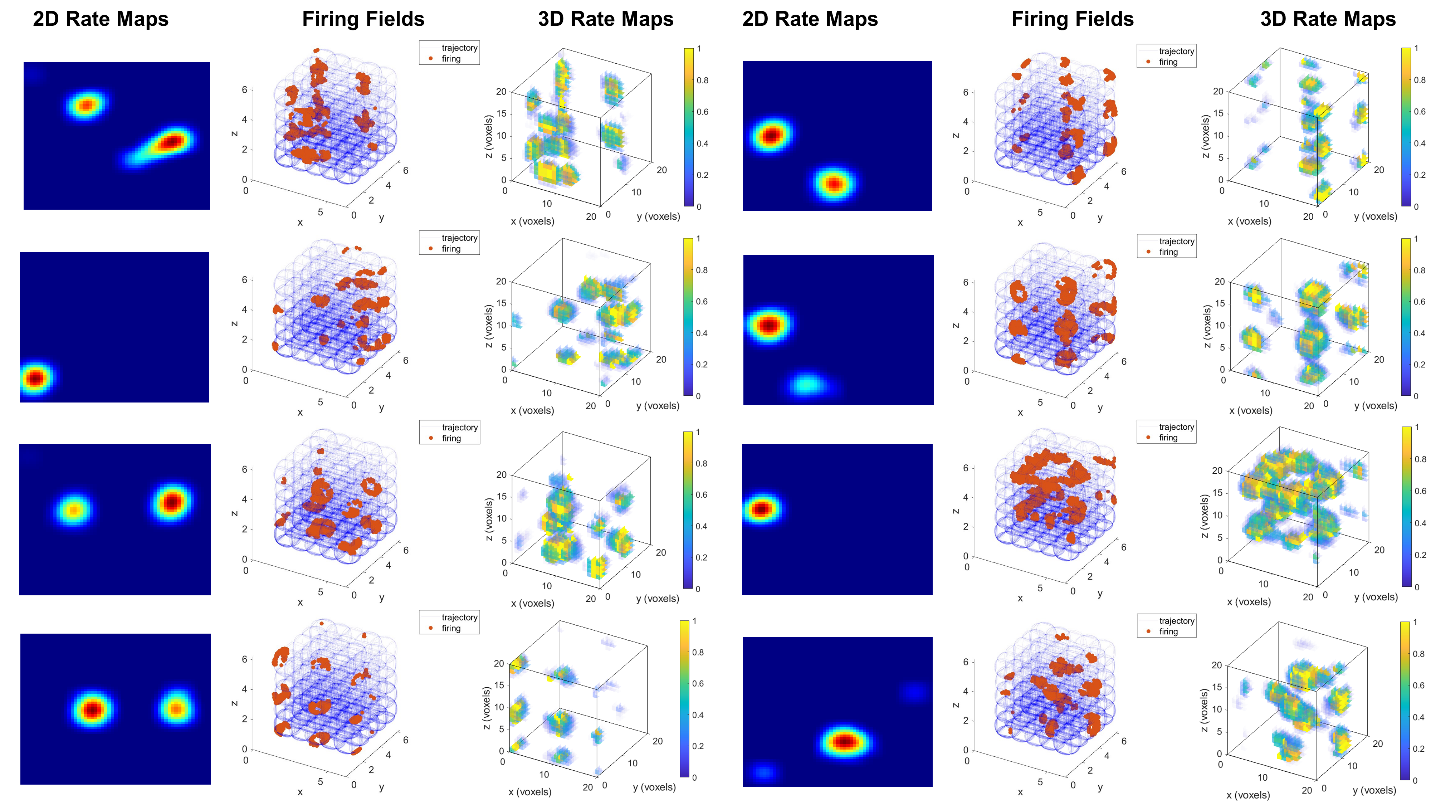


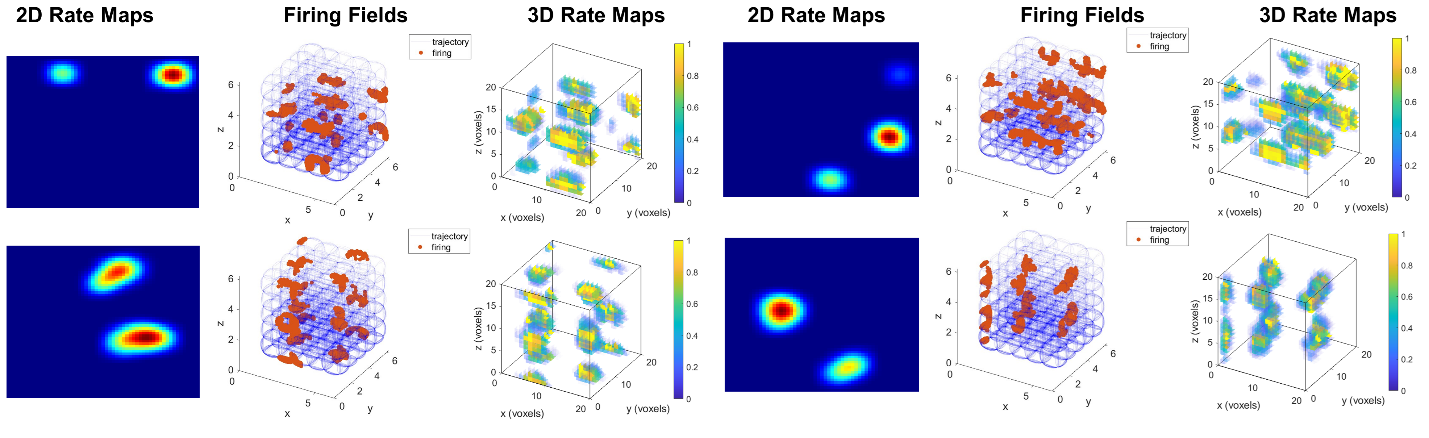


*Figure S1.1: Firing rate maps of all the place cells in 2D arena (1^st^ and 4^th^ columns), firing fields (2^nd^ and 6^th^ columns) and firing rate maps (3^rd^ and 6^th^ columns) of the corresponding place cells in aligned lattice maze.*

*
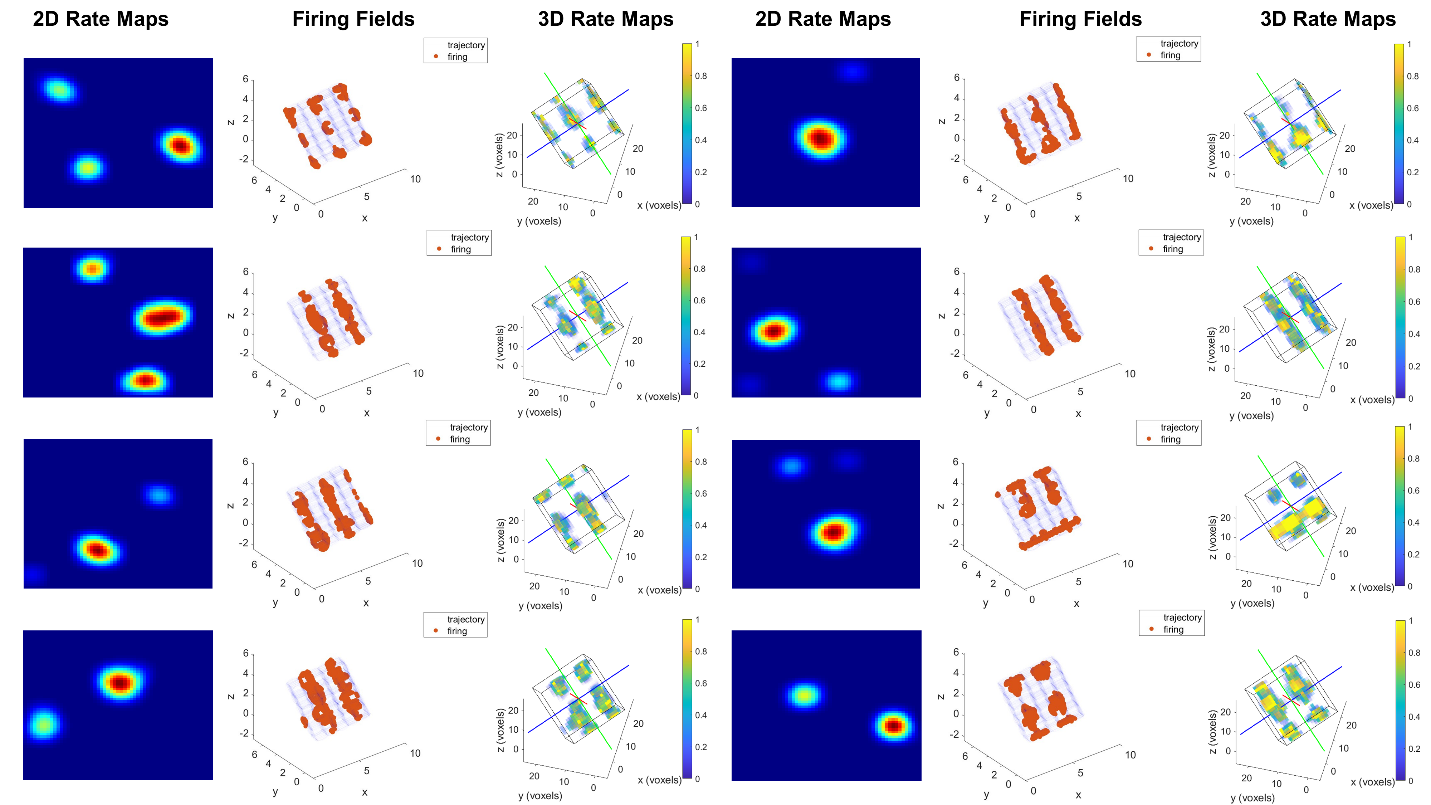
*

*
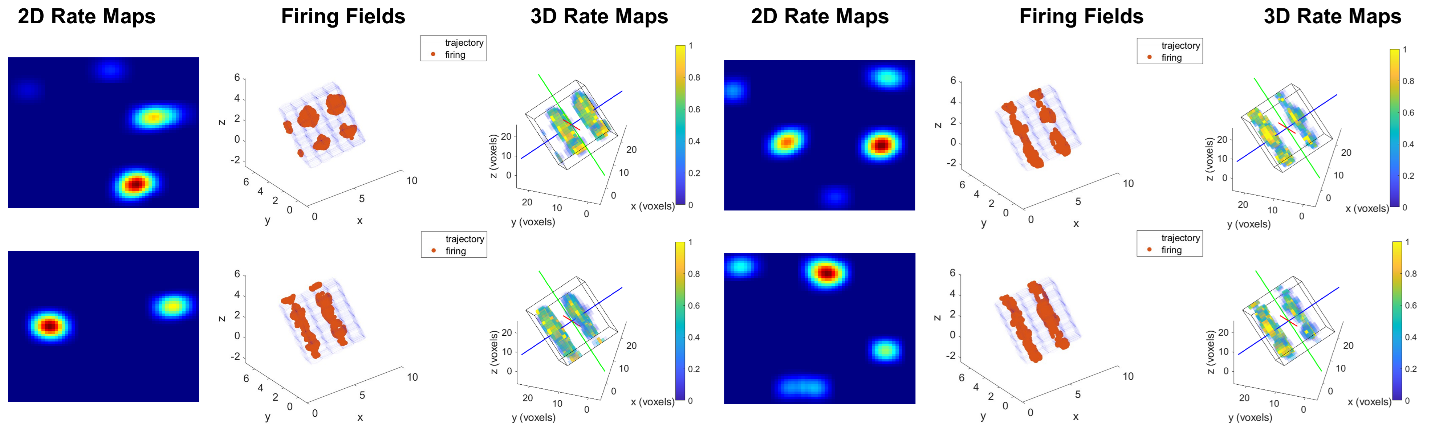
*

*Figure S1.2:* *Firing rate maps of all the place cells in 2D arena (1^st^ and 4^th^ columns), firing fields (2^nd^ and 6^th^ columns) and firing rate maps (3^rd^ and 6^th^ columns) of the corresponding place cells in tilted lattice maze.*

1. Distribution of fields:

We extracted the centroids for all the place fields in the lattice mazes (*regionprops3)* and calculated their medians along each axis. We created a distribution of medians for each axis to test whether the fields were distributed uniformly inside the lattice volume. To achieve this, N random points (equal to the number of fields) were generated and their median was calculated along each axis. This was performed 1000 times for each maze. If the median for a certain axis falls between the 2.5^th^ and 97.5^th^ percentile of the distribution, place fields were considered uniformly distributed along that axis (Fig 4 a3, b3).

1. Field Elongation:

To compare the elongation of the place field for both mazes, we calculated the elongation indices for all place fields. The former was achieved by fitting an ellipsoid around it and calculating the principal axes components for each place field as inspired by (Supplementary material (Grieves et al. 2020)). These principal axes were defined as the major axes of the ellipsoid. Further, an elongation index for each place field is defined as

$$\boldsymbol{Field Elongation Index=}\frac{\boldsymbol{2}\boldsymbol{P}_{\boldsymbol{1}}}{\boldsymbol{P}_{\boldsymbol{2}}\boldsymbol{+}\boldsymbol{P}_{\boldsymbol{3}}}$$

Where *P_1_*, *P_2,_* and *P_3_* are principal axes in descending order. The above elongation index indicates the spherical nature of a field, with an elongation index of 1 indicating a spherical field. Most fields were elongated in both aligned (Mean: 2.00, Std. Error: 0.54) and titled (Mean:1.94, Std. Error: 0.61) lattice.

To test the deviation of the elongation index from a distribution that would be expected by chance, we did the analysis illustrated in the experimental result (Grieves et al. 2020). We created a sphere with the center being the centroid of each place field. The diameter of the sphere is given as

$$\boldsymbol{Equivalent diameter=6}\left( \frac{\boldsymbol{v}_{\boldsymbol{f}}}{\boldsymbol{п}} \right)^{\frac{\boldsymbol{1}}{\boldsymbol{3}}}$$

Where, the trajectories that would lie inside the sphere were screened. A set of normally distributed random points equal to the number of spikes of the place field were generated inside the sphere. The mean of the distribution was the centroid and standard deviation of 1.4 times the equivalent radius. Further each of these points were assigned to a closest point on trajectories screened inside the sphere. This created a normal distribution of spikes inside the sphere. Place fields were identified from this random shuffling of spikes and field elongations were calculated. The shuffling was performed 100 times for each place field. From probability distribution for each place field, we observed whether the elongation index for each field was greater or equal to expected by chance. If the actual elongation fell under the 95th percentile of the distribution, it was considered spherical otherwise non-spherical.

1. Orientation:

To quantify the orientation of place fields, we test whether they were oriented along axes of interest and to differentiate the axes with respect to orientation, we performed the analyses and tests presented in the experimental study (Grieves et al. 2020). The primary principal axis of each place field, also considered as the major axis for the ellipsoid (*field elongation),* was extracted using PCA (*regionprops3*). These vectors were then projected onto a sphere with unit radius on diametrically opposite ends. To test for orientation along a specific axis, we screened the points that fell within those surface regions of the sphere that formed a ~60^o^ cone with apex at origin and axis of interest as cone’ axis. All the points from both directions for each axis were summed and presented as a ratio of total points on the sphere.

To determine whether the axes differed from each other, we calculated 95% confidence intervals for each axis. We sampled a set of orientations for randomly chosen place fields with replacement, such that the set is equal to the total number of fields. This was performed 1000 times for each maze, and ratios for each axis were recalculated. If the proportion of points parallel to a certain axis falls inside the error bars of other axes, they are not considered significantly different. The error bars in (Fig. 6 A3, B3) show the 2.5^th^ and 97.5^th^ percentile for the shuffled counting.

To check whether this ratio for a certain axis was observed more than expected by chance we generated 1000 random points on the surface of the sphere. We calculated the aforementioned proportions for all the axes. We did this 1000 times and calculated the chance as 2.5^th^ and 97.5^th^ percentile of the distribution. The proportion for the axis that exceeded the upper threshold, was considered to have shown significant orientation parallel to a certain axis.

Similar to the experiment study, to check if fields were parallel to XYZ axes in aligned case and to ABC axes in the tilted case, we defined the axis ratio.

Axis Ratio = XYZ / ABC

This ratio was calculated by generating 1000 random points on the surface of a sphere 1000 times. The proportions for each axis were calculated for each shuffle and the distribution for the above ratio was plotted. If the actual ratio deviated from the 1^st^ or 99^th^ percentile, it was considered a significant deviation from the no axis bias condition.

1. Binary Morphology:

The firing rate maps of the place cells are created by first thresholding them at 10% of their maximum value, and a morphological erosion process was performed on the 3D binary volumetric image using structuring element vectors of varying lengths along each cartesian axis. For each erosion operation, the linear sum of the remaining voxels as a measure of the map’s connectivity along that dimension is first calculated and is then expressed as the proportion of all remaining voxels for that element length.

Using this approach, we observed for the aligned lattice that the connectivity of voxel was identical (nearly 33% for all axes) to a specified length of structuring element vector. It started diverging for longer elements, with a larger proportion of fields showing connectivity in the Z-axis compared to others. A repeated measures (within-subjects) ANOVA was run with axes as an independent variable and the proportion of voxels (connectivity) as a dependent variable. The results suggest a significant difference of voxel proportion between all three axes (effect of axes: F (2, 317) = 4.27, p < 0.01, $\boldsymbol{\eta}_{\boldsymbol{p}}^{\boldsymbol{2}}$= 0.026, insignificant effect of axis and length of structuring element: F (36, 317) = 0.878, p = 0.67, $\boldsymbol{\eta}_{\boldsymbol{p}}^{\boldsymbol{2}}$= 0.089). Post hoc tests (Bonferroni correction for pairwise Comparison) for the same revealed difference between voxel proportions for the X-axis (Mean: 0.318, Std. Error: 0.028) and Y-axis (Mean: 0.273, Std. Error: 0.026) as insignificant (did not differ), but significant difference of connectivity with the Z-axis (Mean: 0.409, Std. Error: 0.029). Pairwise comparisons show (X vs Y: p > 0.95, X vs Z: p = 0.22 and Y vs Z: p < 0.01).

Similarly, for the tilted lattice case, the proportion of voxel connectivity stayed equal (nearly 33%) for all the axes. A slight divergence is observed for longer structural elements but is less significant as compared to aligned lattice cases. The similar repeated measures ANOVA test was run with axes as an independent variable and proportion of voxels (connectivity) as a dependent variable. The result suggested a significant difference for voxel proportion between all three axes (effect of axes: F(2, 401) = 51.56, p < 0.0001, $\boldsymbol{\eta}_{\boldsymbol{p}}^{\boldsymbol{2}}$ = 0.199) but with comparatively smaller effect size. For interaction between axis and structure length, the result showed no significant difference (F(35, 401) = 0.822, p = 0.76, $\boldsymbol{\eta}_{\boldsymbol{p}}^{\boldsymbol{2}}$ = 0.066). Post hoc tests (Bonferroni correction for pairwise Comparison) revealed differences between voxel proportions for the A-axis (Mean: 0.306, Std. Error: 0.018), B-axis (Mean: 0.509, Std. Error: 0.020) and C-axis (Mean: 0.184, Std. error: 0.017) as significant difference of connectivity. Pairwise comparisons show (A vs B, B vs C and A vs C: p < 0.0001).

1. Grid and Place cells for helical trajectory:


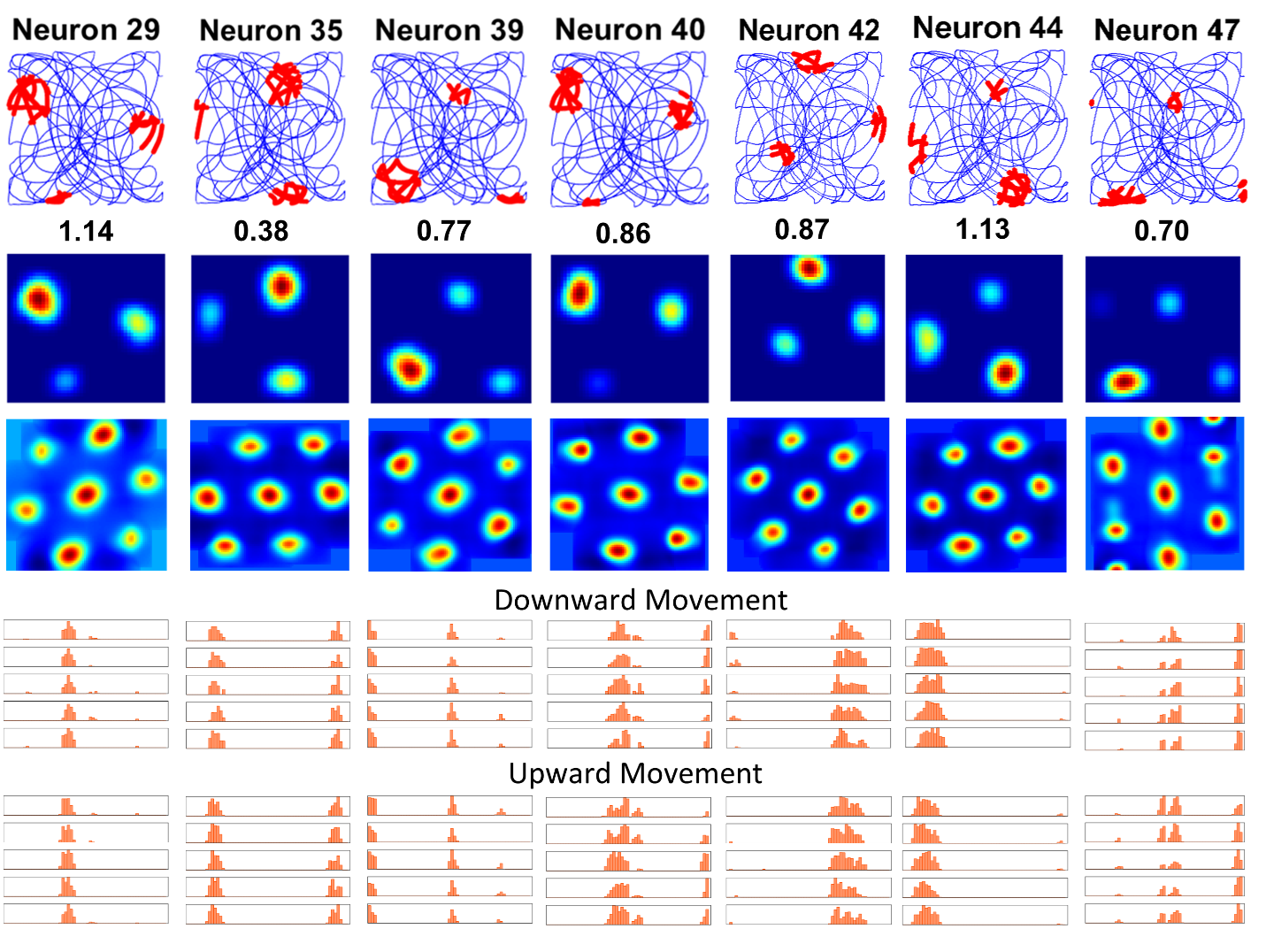


*Figure S2: Grid cells firing fields in flat arena (first row), firing rate maps (second row) and auto correlograms (third row), linearized grid cell firing on helical maze during downward movement (fourth row), linearized grid cell firing on helical maze during upward movement (fifth row) for all the grid cells. The numbers on top of 2^nd^ row i.e., firing rate maps of firing in flat arena are the hexagonal grid scores of the corresponding neurons.*


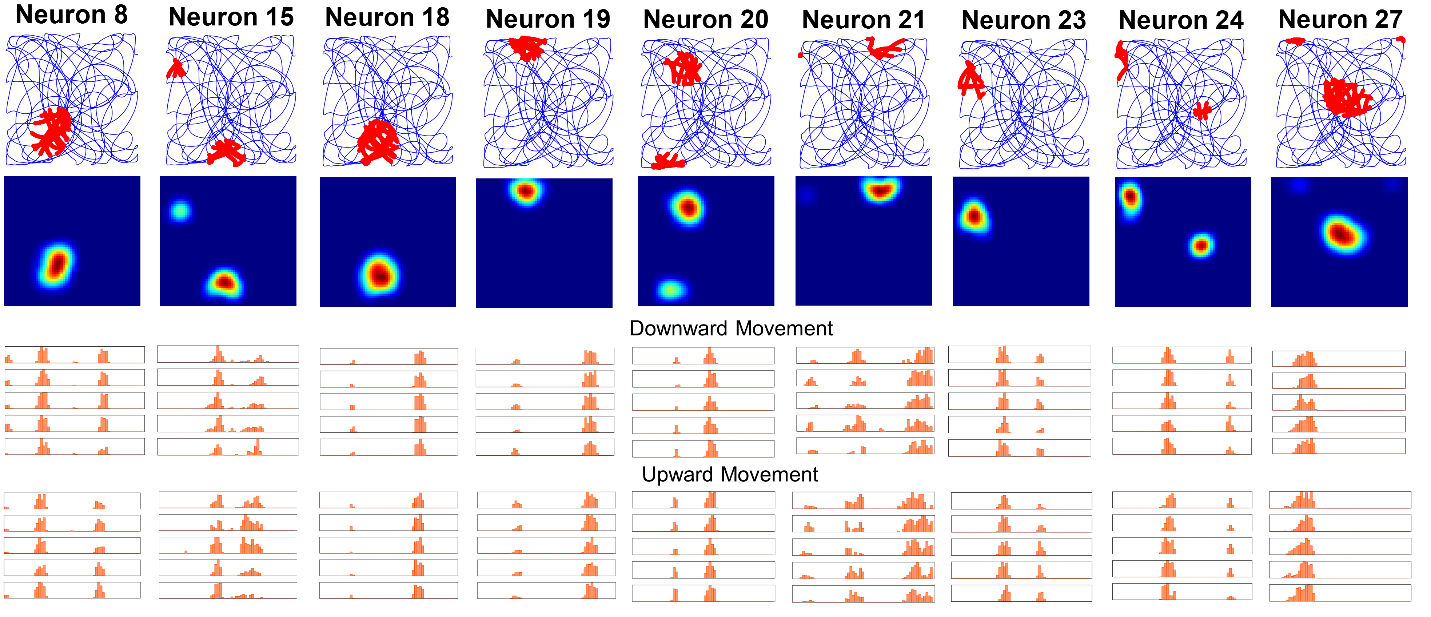


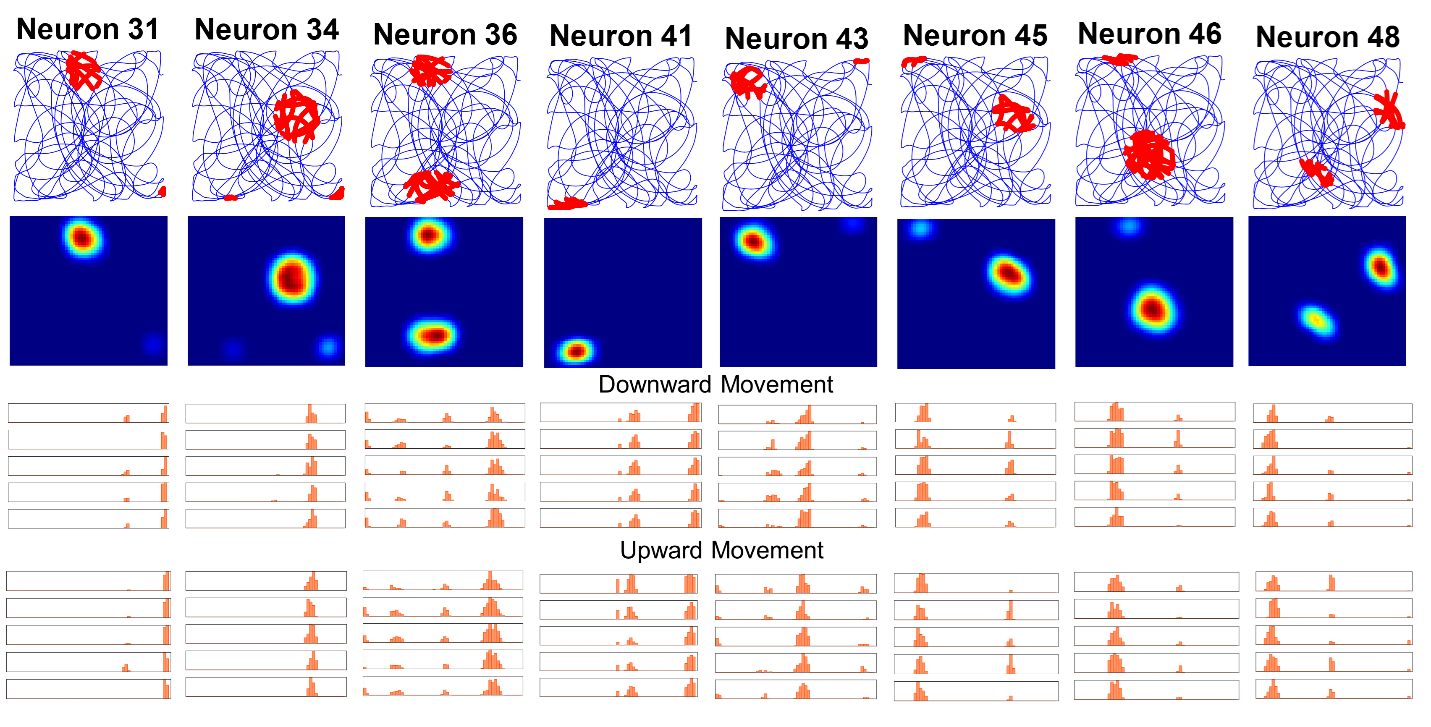


*Figure S3: Place cells firing fields in flat arena (1^st^ row), corresponding firing rate maps (2^nd^ row), linearized firing of corresponding place cells during downward (3^rd^ row) and upward movement (4^th^ row) on helix.*

1. Grid and place cells for Pegboard:


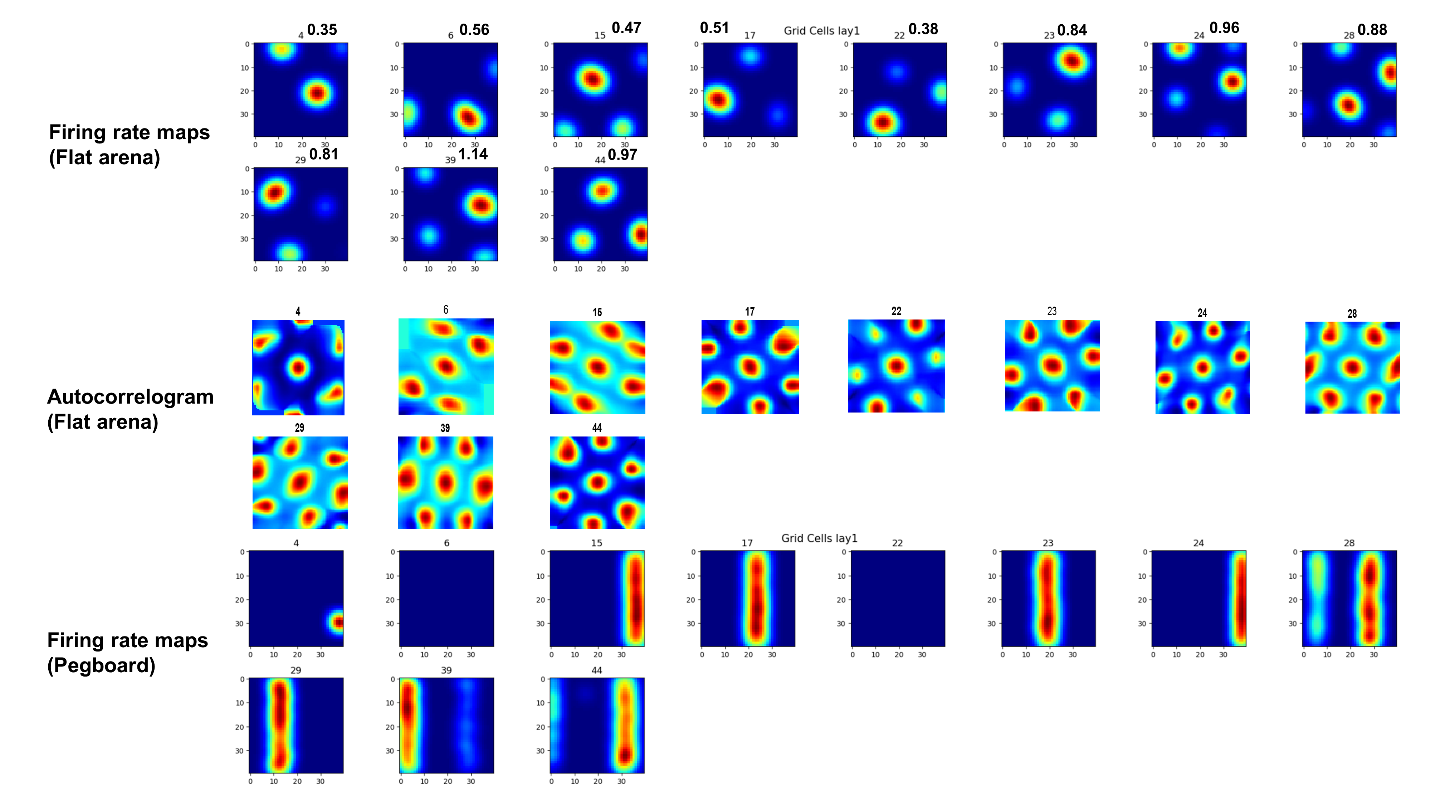


*Figure S4: Grid cells responses. Firing rate maps in flat arena (Top row), Auto correlogram (middle row) and firing rate maps in Pegboard (bottom row).*


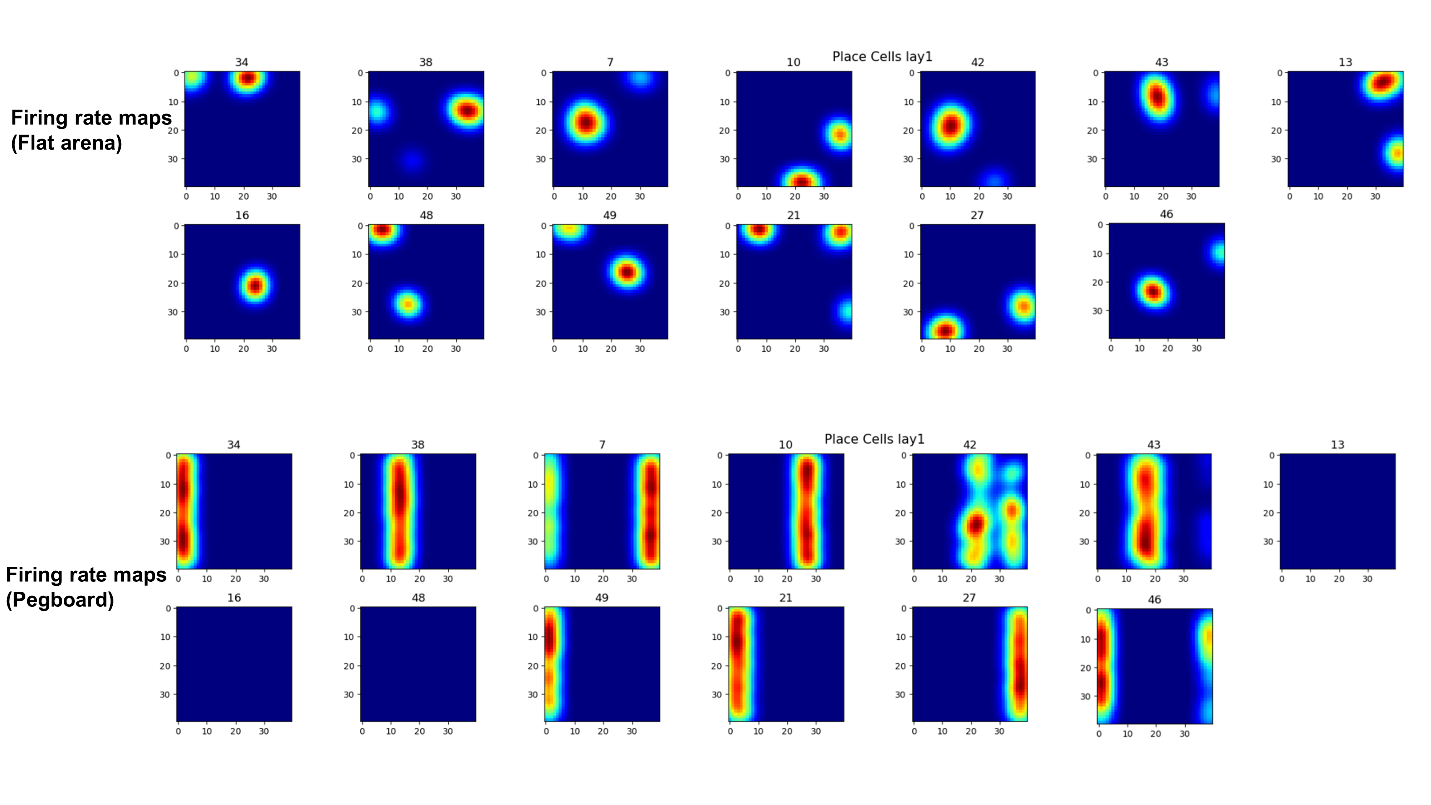


*Figure S5: Place cell responses. Firing rate maps of neurons in Flat arena (upper) and firing rate maps of corresponding neurons in Pegboard (bottom)*

*
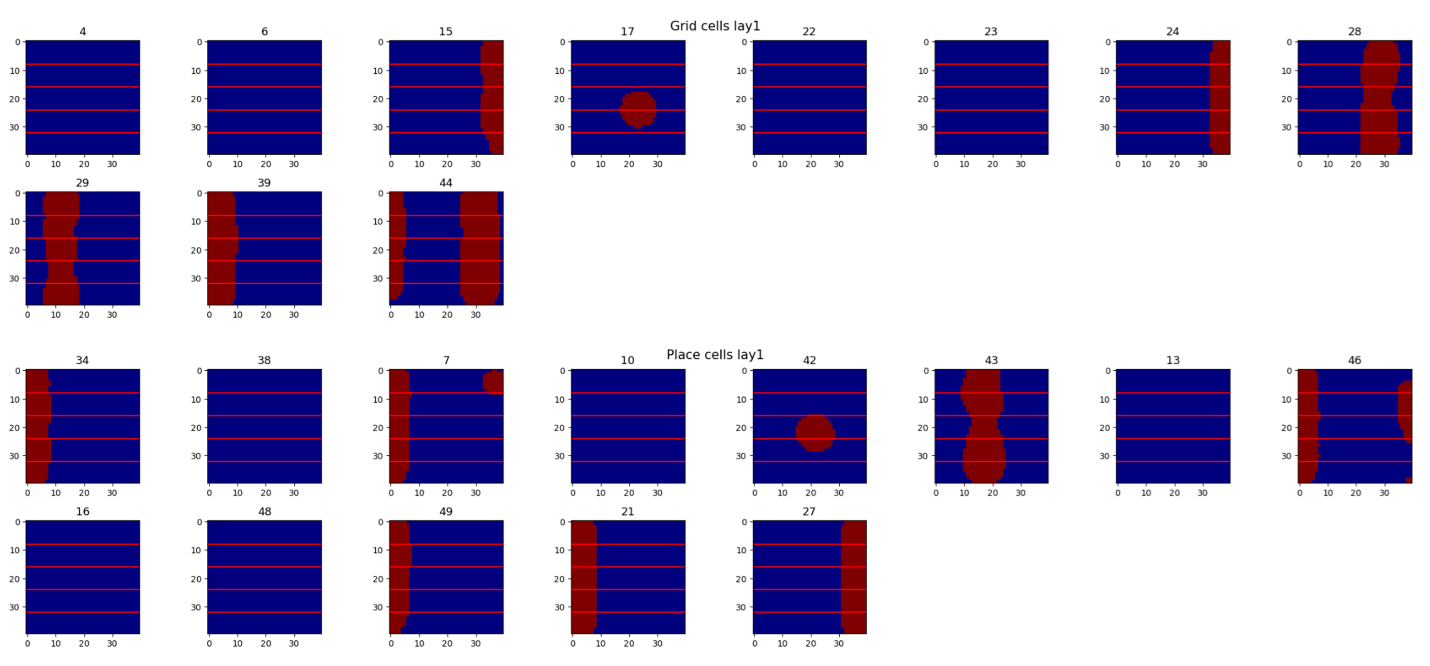
*

*Figure S6: Field expanse for grid (top two rows) and place cell (bottom two rows) responses in the pegboard. The pegboard is divided into 5 equal sectors.*

1. Path Integration (PI) layer: This layer consists of oscillatory neurons that have one-to-one connection with neurons from the HD layer (Soman, Muralidharan, and Chakravarthy 2018). The process involved in this layer can be divided into two major stages. The first stage is frequency modulation of the HD responses. The second stage consists of low pass filtering to eliminate time-dependent high frequency oscillations, which gives the path integration output.

It is evident that theta oscillations exist in the Hippocampal formation and are modulated almost linearly with velocity (Burgess, Barry, and O’Keefe 2007; Geisler et al. 2007). The simplest way to represent frequency is in the form of sinusoids. Hence, we start with sinusoids with theta frequency ($\boldsymbol{\omega)}$ in eqn 1.

The first stage of frequency modulation (FM) of PI is described by the following equations:

$\boldsymbol{PI}_{\boldsymbol{i}_{\boldsymbol{fm}}}\boldsymbol{= sin[\omega t + \beta\int(}\boldsymbol{HD}_{\boldsymbol{i}}\boldsymbol{)dt]}$ (1)

$\boldsymbol{= sin[\omega t + \beta\int(v(t).}\boldsymbol{u}_{\boldsymbol{i}}\boldsymbol{)dt]}$

$\boldsymbol{= sin[\omega t + \beta z.}\boldsymbol{u}_{\boldsymbol{i}}\boldsymbol{]}$ (2)

where, ω is the average base angular frequency of the oscillators which lies in theta frequency (4Hz – 8Hz) observed in the hippocampus, z is the displacement of the animal from its initial position, β is the scaling factor and $\boldsymbol{HD}_{\boldsymbol{i}}$ is the output of Head direction layer (methods section).

This was the model of path integration used in (Soman, Muralidharan, and Chakravarthy 2018; Soman, Muralidharan, and Chakravarthy 2018) (eqn. 1). This is one of the models which overcomes the constraint of specific direction tuning. However, it was also shown that with certain improvements, the OI models can also overcome this problem (Hasselmo and Shay 2014; Kropff and Treves 2008). Note that the PI cell described in eqn. (2) is a spatio-temporal model dependent explicitly on space, z, and on time, t. Such a model was successful in explaining certain temporal phenomena like phase precession (Soman, Muralidharan, and Chakravarthy 2018; Soman, Muralidharan, and Chakravarthy 2018). However, most spatial cells are described in purely spatial terms (e.g., place cells and grid cells), depicting their responses as exclusive functions of space. In the present study, we are interested in describing these purely spatial responses. Therefore, we convert the spatio-temporal model of eqn. (2) into a purely spatial model by an averaging process described below.

In order to eliminate temporal variation, a sin ($\boldsymbol{\omega t}$) term is multiplied with the $\boldsymbol{PI}_{\boldsymbol{i}_{\boldsymbol{fm}}}$ shown in eqn. 2, and passed through a low pass filter which blocks the high frequency signals as shown in eqn. 3 and eqn. 4.

$\boldsymbol{P}\boldsymbol{I}_{\boldsymbol{i}}\boldsymbol{=sin}\left( \boldsymbol{\omega t} \right)\boldsymbol{sin}\left[ \boldsymbol{\omega t+\beta z.}\boldsymbol{u}_{\boldsymbol{i}} \right]$ (3)

$\boldsymbol{P}\boldsymbol{I}_{\boldsymbol{i}}\boldsymbol{=cos}\left( \boldsymbol{\beta z.}\boldsymbol{u}_{\boldsymbol{i}} \right)\boldsymbol{-cos(2}\boldsymbol{\omega t+}$ $\boldsymbol{\beta z.}\boldsymbol{u}_{\boldsymbol{i}}$)

After eliminating the high frequency term, we have,

$\boldsymbol{P}\boldsymbol{I}_{\boldsymbol{i}}\boldsymbol{\approx}\boldsymbol{cos}\left( \boldsymbol{\beta z.}\boldsymbol{u}_{\boldsymbol{i}} \right)$ (4)

1. Grid cell distributions:


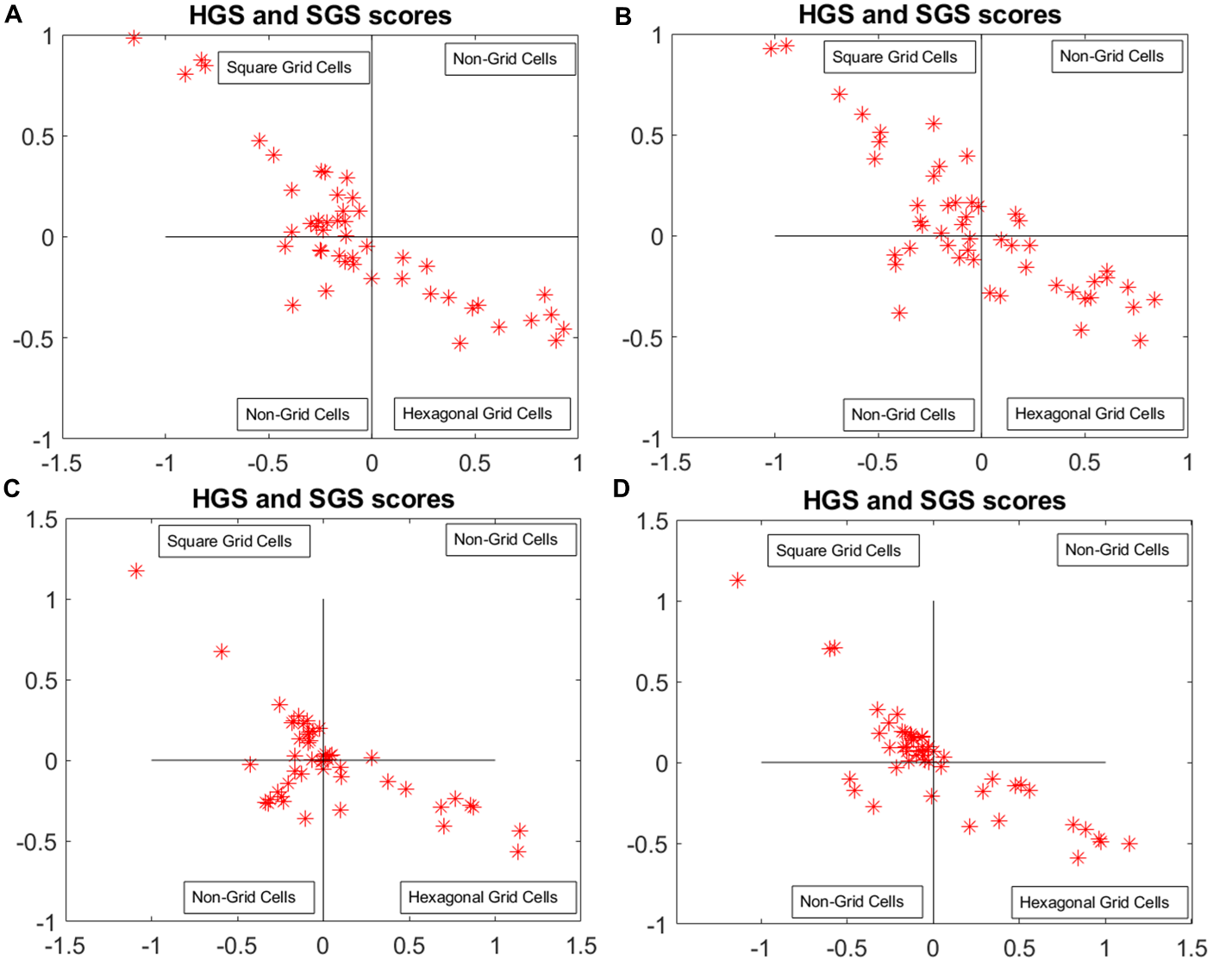


*Figure S7: Distribution of 50 neurons in their respective categories. 2^nd^ and 4^th^ quadrants account for square grid cells and hexagonal grid cells, although, neurons with hexagonal grid scores > 0.3 are considered as hexagonal grid cells. A) shows for aligned lattice maze, B) shows for tilted lattice maze, C) shows for helix and D) shows for pegboard*.

References

Burgess, Neil, Caswell Barry, and John O’Keefe. 2007. “An Oscillatory Interference Model of Grid Cell Firing.” *Hippocampus* 17(9):801–12.

Geisler, Caroline, David Robbe, Michaël Zugaro, Anton Sirota, and György Buzsáki. 2007. “Hippocampal Place Cell Assemblies Are Speed-Controlled Oscillators.” *Proceedings of the National Academy of Sciences of the United States of America* 104(19):8149–54.

Grieves, Roddy M., Selim Jedidi-Ayoub, Karyna Mishchanchuk, Anyi Liu, Sophie Renaudineau, and Kate J. Jeffery. 2020. “The Place-Cell Representation of Volumetric Space in Rats.” *Nature Communications 2020 11:1* 11(1):1–13.

Hasselmo, Michael E., and Christopher F. Shay. 2014. “Grid Cell Firing Patterns May Arise from Feedback Interaction between Intrinsic Rebound Spiking and Transverse Traveling Waves with Multiple Heading Angles.” *Frontiers in Systems Neuroscience* 8(October):201.

Kropff, Emilio, and Alessandro Treves. 2008. “The Emergence of Grid Cells: Intelligent Design or Just Adaptation?” *Hippocampus* 18(12):1256–69.

Soman, Karthik, Vignesh Muralidharan, and V. Srinivasa Chakravarthy. 2018. “A Model of Multisensory Integration and Its Influence on Hippocampal Spatial Cell Responses.” *IEEE Transactions on Cognitive and Developmental Systems* 10(3):637–46.

Soman, Karthik, Vignesh Muralidharan, and Vaddadi Srinivasa Chakravarthy. 2018. “A Unified Hierarchical Oscillatory Network Model of Head Direction Cells, Spatially Periodic Cells, and Place Cells.” *European Journal of Neuroscience* 47(10):1266–81.

.
